# Green and Emerging Microextraction Strategies for Bioanalytical Determination of Hormones: Trends, Challenges, and Applications

**DOI:** 10.3390/molecules30224471

**Published:** 2025-11-19

**Authors:** David Vicente-Zurdo, Sonia Morante-Zarcero, Isabel Sierra

**Affiliations:** 1 Departamento de Tecnología Química y Ambiental, Escuela Superior de Ciencias Experimentales y Tecnología (E.S.C.E.T), Universidad Rey Juan Carlos, C/Tulipán s/n, 28933 Móstoles, Spain; david.vicente.zurdo@urjc.es; 2 Instituto de Investigación de Tecnologías para la Sostenibilidad, Universidad Rey Juan Carlos, C/Tulipán s/n, 28933 Móstoles, Spain

**Keywords:** bioanalytical matrices, green analytical chemistry, green solvents, liquid chromatography, mass spectrometry, microextraction techniques, smart materials, steroidal hormones, thyroid hormones

## Abstract

Accurate and sensitive determination of hormones in biological matrices is essential for clinical diagnostics, therapeutic monitoring, and endocrine research. However, hormone determination presents significant challenges due to their typically low concentrations, complex sample matrices, and structural diversity. In recent years, microextraction techniques have emerged as strategic tools in bioanalytical chemistry, offering advantages in terms of miniaturization, enhanced selectivity, and compatibility with the principles of green analytical chemistry (GAC). This review provides a comprehensive overview of green and emerging microextraction approaches for the determination of steroidal, thyroid, peptide, and other hormones in biological samples. Key techniques such as solid-phase microextraction (SPME) and dispersive liquid–liquid microextraction (DLLME), followed by high-performance liquid chromatography (HPLC) coupled to diode array detectors (DADs) or mass spectrometry (MS), are critically discussed. Special emphasis is placed on the use of environmentally friendly solvents, such as deep eutectic solvents (DESs), supramolecular solvents (SUPRASs), and advanced sorbents including molecularly imprinted polymers (MIPs) and nanostructured magnetic phases. Applications across various bioanalytical matrices (urine, plasma, serum, saliva, tissues…) are examined in terms of sensitivity, selectivity, and validation parameters. Finally, current challenges, method development gaps, and future directions are highlighted to support the continued advancement of sustainable hormone determination in complex biological systems.

## 1. Introduction

Hormones are pivotal regulators of biological function, orchestrating essential physiological processes such as growth, metabolism, reproduction, and homeostasis [1]. The ability to accurately quantify hormones in biological matrices is critical across a range of bioanalytical contexts, including clinical diagnostics [2], endocrine disruption studies [3], therapeutic drug monitoring [4], sports anti-doping programs [5], and pharmacokinetic evaluations [6]. Given the tight hormonal regulation in the human body, even slight fluctuations in hormone levels can reflect pathological conditions or physiological imbalances [7]. However, the quantification of hormones remains analytically demanding due to their occurrence at ultra-trace levels, often in the ng L^−1^ to pg L^−1^ range, within complex biological matrices [7]. These concentration levels can vary substantially depending on the hormone family, bioanalytical matrix, and physiological condition of the individual. For instance, steroidal hormones are typically found in the low ng L^−1^ range in serum and urine, whereas peptide hormones may occur at higher but still trace levels. This variability underscores the analytical challenge of achieving sufficient sensitivity and selectivity across different hormone families and sample types. These samples typically contain a variety of potentially interfering substances, including proteins, lipids, salts, and endogenous metabolites, which can compromise analytical selectivity and sensitivity [8]. As a result, robust, efficient, and selective sample preparation strategies are essential to isolate target hormones and minimize matrix effects prior to instrumental analysis.

In recent decades, conventional sample preparation techniques such as liquid–liquid extraction (LLE), solid-phase extraction (SPE), and protein precipitation have been routinely employed for hormone determination in biological matrices. Although these methods have proven effective, they often require large sample and solvent volumes, involve multiple manual steps, and generate considerable chemical waste, which limits their sustainability and miniaturization potential. To overcome these drawbacks, microextraction techniques have emerged as strategic tools in overcoming these challenges, particularly in the context of modern analytical demands that prioritize green analytical chemistry (GAC), miniaturization, and enhanced analytical performance [9]. Compared to classical techniques, microextraction formats (including solid-phase microextraction (SPME), dispersive liquid–liquid microextraction (DLLME), supramolecular solvent (SUPRAS) systems, and deep eutectic solvent (DES/NADES)-based approaches) achieve equivalent or superior analytical performance while drastically reducing solvent consumption, sample handling, and analysis time. This evolution represents a significant methodological shift towards greener and more sustainable bioanalytical workflows. These techniques rely on minimal or no use of organic solvents, reducing both environmental impact and operator exposure, and are compatible with a broad spectrum of analytical separation techniques such as gas chromatography (GC) [10], liquid chromatography (LC) [11] or capillary electrophoresis (CE) [12], and also with mass spectrometry (MS) analyzers [13]. The integration of microextraction with advanced materials, such as nanostructured sorbents and molecularly imprinted polymers (MIPs), has further enhanced their selectivity toward specific hormonal families [14]. In addition, microextraction enables the preconcentration of analytes, effectively boosting detection sensitivity while reducing the need for complex, time-consuming workflows. As regulatory agencies and scientific communities increasingly emphasize sustainable and high-throughput analytical solutions, microextraction methods are gaining prominence as front-line tools for hormone monitoring in biological samples.

This review provides a comprehensive overview of green and emerging microextraction strategies specifically applied to the bioanalytical determination of hormones. The microextraction-based analytical methods discussed in this review have demonstrated adequate sensitivity to reliably quantify hormones within ultra-trace concentration ranges. The scope includes a classification of hormones of interest (steroidal, thyroid, peptide and protein, among others) alongside a discussion of their analytical significance [15]. An in-depth examination of the main microextraction techniques follows, including SPME [16], DLLME [17], and stir bar sorptive extraction (SBSE) [18], as well as novel and hybrid methods such as in vivo SPME [19] and solvent bar microextraction (SBME) [20]. Special emphasis is placed on the use of green solvents, namely DES, NADES, and SUPRAS [21], and smart materials such as MIPs, magnetic nanoparticles (MNPs), and other nanostructured sorbents that enhance the selectivity and sustainability of the extraction process [22]. The application of these techniques across a wide range of biological matrices is critically examined, with a focus on analytical performance indicators such as sensitivity, selectivity and robustness. The review culminates in the identification of current gaps in method development and standardization. Finally, future perspectives are presented, highlighting emerging trends, the application of chemometrics in method optimization, advances in green materials, and the progress toward regulatory harmonization in the determination of hormones in complex biological environments.

### Search Strategy and Inclusion Criteria

In line with internationally recognized standards for high-quality review studies, this work is defined as a narrative critical review with systematic search elements. Its primary objective is to identify and classify recent eco-friendly and miniaturized microextraction strategies applied to the bioanalytical determination of hormones in biological matrices, while critically evaluating their analytical performance, green attributes, and practical applicability. Although not a systematic review, the present study incorporates transparent search and selection procedures to enhance reproducibility and methodological rigor. This approach enables a comprehensive and critical synthesis of current advances, challenges, and future directions in sustainable microextraction for hormone determination.

The selection of relevant studies was primarily carried out using Scopus and ScienceDirect as the main reference databases. The search strategy employed the following detailed Boolean string “hormone AND microextraction AND (SPME OR LPME OR SBSE OR SBME OR DES OR NADES OR MIPS OR (bioanalytical AND matrix))”. The search covered the period from January 2020 to June 2025, with the final search conducted on 24 June 2025. Inclusion and exclusion criteria were clearly defined to retain only studies focusing on the bioanalytical determination of hormones using green or miniaturized microextraction techniques. Inclusion criteria were restricted to articles published in English, and duplicate records were removed to prevent redundancy. Conversely, works that did not involve biological matrices were excluded. To enhance methodological transparency and minimize selection bias, two independent authors conducted the screening and data extraction processes. Each record identified through the database search was independently evaluated according to predefined inclusion and exclusion criteria. Any discrepancies or uncertainties between the two independent authors were resolved through discussion with a third author until consensus was reached.

**Figure 1:**
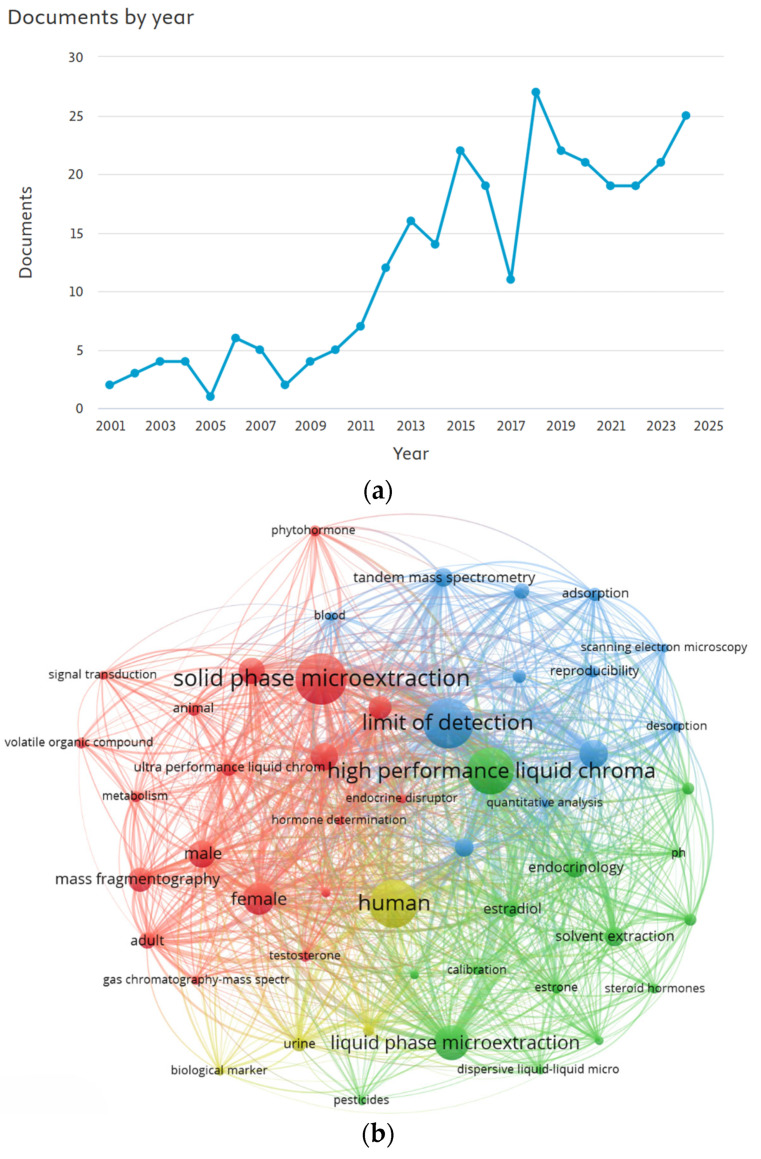
(**a**) Number of documents per year retrieved from Scopus using the keyword strategy outlined in the methodology; (**b**) Keyword co-occurrence map generated using VOSviewer (version 1.6.20), based on 119 Scopus-indexed documents related to this study published between 2020 and 2025.

Data retrieved from Scopus highlight a steady increase in the number of publications on this topic since 2001 (Figure 1a). Additionally, the keyword co-occurrence map (Figure 1b) reveals strong associations with terms such as “solid phase microextraction”, “high performance liquid chromatography”, “limit of detection”, and “human”. This network visualization illustrates the conceptual interconnectivity and thematic clustering among these terms, reflecting a growing interdisciplinary interest in hormone microextraction from bioanalytical matrices within the broader context of green analytical chemistry.

## 2. Hormones Classification and Analytical Relevance

**Figure 2:**
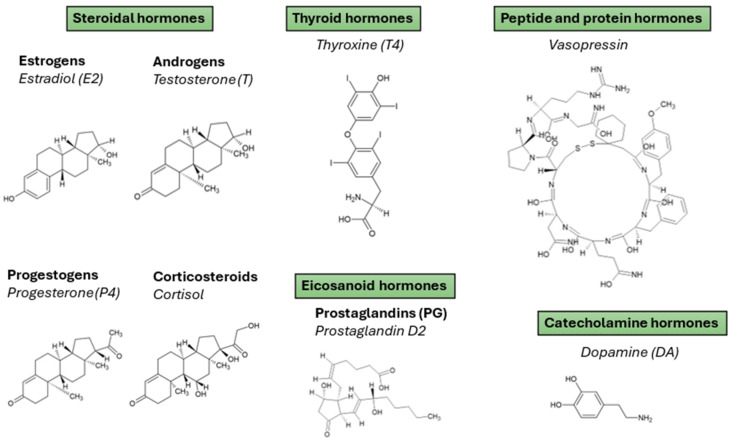
Hormones classification according to their structural family with some representative examples from each family.

Hormones are essential biochemical messengers involved in the regulation of diverse physiological processes, including growth, metabolism, reproduction, and homeostasis. From a bioanalytical perspective, hormones of interest can be broadly classified into four major categories based on their chemical structure and biological function: steroidal hormones, thyroid hormones (THs), peptide/protein hormones, and others (including catecholamine and eicosanoids) (Figure 2). These classes encompass a variety of clinically and environmentally relevant analytes whose trace-level determination in biological matrices remains analytically challenging and highly relevant.

### 2.1. Steroidal Hormones

Steroidal hormones constitute the most extensively studied group due to their widespread physiological roles and implications in disorders such as cancer, endocrine disruption, and reproductive dysfunction. Steroidal hormones are synthesized primarily in endocrine glands such as the adrenal cortex, ovaries, testes, and placenta, and exert their functions through specific intracellular receptors that regulate gene transcription. Based on their biological activity and site of synthesis, these hormones can be broadly classified into four main groups, such as estrogens (estrone (E1), estradiol (E2), estriol (E3), ethinylestradiol (EE2), etc.) [23], androgens (testosterone (T), dihydrotestosterone (DHT), androsterone, dehydroepiandrosterone (DHEA), androstenedione, etc.) [24], progestogens (progesterone (P4), 17-hydroxyprogesterone (17-OHP), pregnenolone, etc.) [25] and corticosteroids (cortisol, hydroxycortisol, cortisone, aldosterone, etc.) [26].

Estrogens are primarily involved in the regulation of female reproductive physiology. The main endogenous estrogens include E1, E2, and E3, with EE2 commonly used in oral contraceptives and hormone replacement therapies. These hormones are mainly produced in the ovaries, although peripheral conversion in adipose tissue also contributes to their levels. Estrogens modulate the menstrual cycle, influence secondary sexual characteristics, and play roles in bone metabolism, cardiovascular function, and neuroprotection. Their concentrations fluctuate throughout the menstrual cycle and pregnancy, and they are commonly monitored in serum, urine, and occasionally in saliva and hair for diagnostic purposes [23].

Androgens are key regulators of male reproductive development and function but are also present in females at lower concentrations. T is the principal androgen, synthesized in the Leydig cells of the testes and, to a lesser extent, in the adrenal cortex and ovaries. Other biologically active androgens include DHT, androstenedione, DHEA, and androsterone. These hormones regulate spermatogenesis, libido, muscle mass, and the development of male secondary sex characteristics. Abnormal androgen levels are associated with disorders such as polycystic ovary syndrome, hypogonadism, and androgenic alopecia [24]. Due to their low levels and structural similarity, their detection in biological matrices such as urine, serum, or plasma requires high specificity.

Progestogens, with P4 being the most significant member, are crucial for maintaining pregnancy and regulating the menstrual cycle. P4 is produced primarily by the corpus luteum and the placenta, with minor contributions from the adrenal glands. It prepares the endometrium for embryo implantation, supports gestation, and modulates immune responses during pregnancy. 17-OHP and pregnenolone are additional progestogenic steroids involved in the biosynthetic pathway of other hormones. Monitoring P4 and its derivatives in serum or urine is essential in fertility assessments, luteal phase deficiency diagnosis, and congenital adrenal hyperplasia screening [25,27].

Corticosteroids are divided into glucocorticoids and mineralocorticoids, both synthesized in the adrenal cortex. Glucocorticoids, such as cortisol and cortisone, regulate carbohydrate metabolism, immune responses, and stress adaptation. Mineralocorticoids, including aldosterone, are involved in electrolyte balance and blood pressure regulation. Dysregulation of corticosteroid levels can indicate endocrine disorders such as Addison’s disease, Cushing’s syndrome, or congenital adrenal hyperplasia. These hormones are typically analyzed in blood, urine, saliva, and even hair to assess both acute and chronic exposure [28,29].

Steroidal hormones are present in a variety of biological matrices depending on the purpose of analysis. While blood and serum are commonly used for real-time physiological assessments, matrices like urine and saliva provide non-invasive alternatives for short-term monitoring. Additionally, keratinized matrices such as hair and nails are gaining interest for retrospective or long-term exposure assessments [30]. The choice of matrix often depends on the hormone’s physicochemical properties, the temporal window of interest, and the clinical or toxicological context. Their lipophilic nature, low endogenous concentrations, and structural similarity require highly selective and sensitive analytical methods, reflecting their clinical relevance and the need for green analytical methodologies.

### 2.2. Thyroid Hormones

THs, primarily thyroxine (T4), triiodothyronine (T3), and the less abundant diiodothyronine (T2), constitute a distinct class of iodinated organic compounds derived from the amino acid tyrosine. Synthesized and secreted by the thyroid gland, these hormones play a fundamental role in regulating basal metabolic rate, thermogenesis, growth, and neurological maturation. Their classification is based on the degree of iodination: T4 contains four iodine atoms, T3 contains three, and T2 contains two, with further distinctions made between positional isomers such as 3,3′-T2 and 3,5-T2. Additionally, reverse T3 (rT3), an inactive isomer of T3, is a relevant analytical target in certain diagnostic contexts.

From a biochemical standpoint, THs are unique among endocrine analytes due to their amphipathic nature and extensive binding to plasma proteins, including T4-binding globulin, transthyretin, and albumin. This strong affinity complicates the selective quantification of free (biologically active) versus total hormone levels. Since only a small fraction of THs circulates in the unbound state, sensitive and specific analytical methods are required to assess physiological and pathological alterations accurately.

Analytically, THs are of high interest not only in clinical endocrinology but also in environmental health, toxicology, and biomonitoring studies, where they are increasingly used as biomarkers of endocrine disruption due to their sensitivity to environmental contaminants [31]. In clinical diagnostics, the precise determination of T3, T4, rT3, and their ratios (T3/T4, rT3/T3, for example) is essential for evaluating thyroid function, diagnosing disorders such as hypothyroidism and hyperthyroidism, and guiding hormone replacement therapy. In occupational and environmental health contexts, THs are increasingly recognized as sentinel biomarkers of endocrine disruption, due to their sensitivity to xenobiotic interference and their systemic regulatory roles.

The analytical relevance of THs is further amplified by their structural similarity and chemical instability under certain conditions. Their determination often requires pre-analytical steps that ensure stabilization, deproteinization, or derivatization. Additionally, distinguishing between endogenous hormone levels and those introduced exogenously (from medications or contaminated supplements, for example) requires methods with both high selectivity and robust quantification capabilities.

From the perspective of bioanalytical method development, THs pose several key challenges, such as low endogenous concentrations (particularly in free hormone forms), high matrix complexity (especially in blood, serum, urine, and keratinized matrices such as hair), interference by structurally related iodinated compounds or metabolites and requirement for accurate differentiation between isomers (T3 and rT3), which has prompted the development of signal enhancement strategies and micellar electrophoretic techniques tailored for THs in complex matrices [32].

These challenges have driven the development of increasingly sophisticated analytical approaches, including microextraction techniques coupled with high-resolution separation and detection platforms. The successful determination of THs thus represents a critical benchmark for method sensitivity, selectivity, and overall analytical performance, particularly in matrices where only trace levels are present.

In summary, THs form a chemically well-defined yet analytically complex group of biomarkers whose quantification is central to both clinical endocrinology and broader applications in environmental and biomedical research. Their physicochemical characteristics and physiological roles make them demanding yet informative targets for modern microextraction-based analytical methodologies.

### 2.3. Peptide and Protein Hormones

Peptide and protein hormones constitute a diverse class of biomolecules characterized by their polypeptidic nature and high molecular weight compared to steroidal hormones or THs. These hormones are synthesized as precursor proteins and undergo extensive post-translational modifications before being secreted into circulation. Their water solubility eliminates the need for carrier proteins but also poses specific analytical challenges due to their susceptibility to enzymatic degradation and structural complexity.

These hormones play essential roles in regulating numerous physiological processes including metabolism, growth, reproduction, stress response, and lactation. Based on their structure and function, peptide and protein hormones can be broadly classified into: (i) hypothalamic and neurohypophyseal hormones (such as oxytocin or vasopressin), (ii) pituitary hormones (such as luteinizing hormone (LH), follicle-stimulating hormone (FSH), growth hormone (GH) or prolactin (PRL)), (iii) metabolic hormones (such as insulin), and (iv) emerging neuroendocrine peptides (such as kisspeptin).

Neurohypophyseal peptides such as oxytocin and vasopressin are nonapeptides produced in the hypothalamus and released from the posterior pituitary. Oxytocin is involved in parturition and lactation, whereas vasopressin regulates water homeostasis and blood pressure. Despite their relatively small size among peptide hormones, their polar nature and structural instability challenge their bioanalytical determination.

Anterior pituitary hormones, including LH, FSH, GH, and PRL, are glycoproteins or polypeptides with central roles in endocrine feedback loops. LH and FSH, often referred to as gonadotropins, are critical regulators of the reproductive axis, mediating ovarian follicular development and spermatogenesis. Their levels are clinically relevant in fertility assessment, pubertal disorders, and the evaluation of endocrine-disrupting chemical (EDC) exposure. For example, Brinke et al. examined the genetic background of endocrine fertility parameters, including LH and FSH, in swine models and discussed their role in reproductive outcomes [33]. GH, a single-chain protein hormone, is primarily involved in growth and metabolic regulation, while PRL modulates lactation and reproductive behavior.

Insulin, secreted by pancreatic β-cells, is the prototypical metabolic peptide hormone and a key biomarker in diabetes and metabolic syndrome. Its low endogenous concentrations, extensive degradation by proteases, and high affinity for receptors necessitate sensitive and selective analytical methods for accurate quantification in clinical and research settings.

Kisspeptin, a relatively recent addition to the family of neuroendocrine hormones, has garnered increasing interest for its upstream role in the hypothalamic-pituitary-gonadal axis. It modulates gonadotropin-releasing hormone (GnRH) secretion and is pivotal in regulating puberty onset, reproductive function, and fertility. Its growing relevance has positioned it as a potential biomarker for reproductive toxicity and EDC exposure, as demonstrated in adolescent population studies.

From an analytical standpoint, peptide and protein hormones exhibit distinctive behavior compared to low-molecular-weight compounds. Their larger size, conformational variability, and low abundance in biological matrices necessitate the use of highly selective and robust methodologies. Unlike steroidal hormones, which can often be analyzed using GC or LC with minimal derivatization, peptide hormones typically require immunoaffinity enrichment, proteolytic digestion, or advanced bioanalytical tools such as MS coupled to targeted extraction methods.

Moreover, their bioanalytical relevance is expanding beyond classical endocrinology into environmental health, toxicology, and developmental biology. For example, serum kisspeptin and gonadotropin levels (LH and FSH) are sensitive to low-dose chemical mixtures in adolescent males, highlighting the importance of monitoring these peptides in human biomonitoring programs [34].

To conclude, peptide and protein hormones form a biochemically and functionally heterogeneous group of analytes whose clinical and toxicological importance is growing. Their complex structure and low physiological concentrations pose significant analytical challenges, particularly in the context of non-invasive biomonitoring and EDC risk assessment. As interest increases in hormones like kisspeptin, LH, FSH, and insulin, method development efforts must prioritize strategies capable of achieving high sensitivity and specificity while accommodating the unique biochemical properties of these macromolecules [24].

### 2.4. Other Hormones

In addition to the well-characterized classes of steroidal, thyroid, and peptide/protein hormones, several other hormonally active molecules play critical physiological roles and hold increasing relevance in bioanalytical contexts. Among these, catecholamines and their metabolites, as well as eicosanoids such as prostaglandins (PGs), deserve particular attention due to their unique mechanisms of action, chemical properties, and analytical challenges.

Catecholamines, including dopamine (DA), epinephrine (also known as adrenaline, E), and norepinephrine (NE), are derived from the amino acid tyrosine and function primarily as neurotransmitters and hormones in the sympathetic nervous system. These compounds are crucial for the regulation of cardiovascular function, stress response, and central nervous system (CNS) signaling. Due to their rapid metabolism and low circulating concentrations, the quantification of catecholamines in biological fluids-typically urine, plasma, or serum-requires highly sensitive and selective analytical methods. Their labile and polar nature adds to the complexity of sample preparation and detection. Importantly, their major metabolites, metanephrine and normetanephrine, are formed via catechol-O-methyltransferase-mediated methylation and serve as stable biomarkers for the diagnosis of pheochromocytoma and related adrenal disorders. The clinical and diagnostic significance of these analytes has driven the development of tailored microextraction strategies, such as microextraction by packed sorbent (MEPS), to achieve rapid and efficient preconcentration prior to high-performance liquid chromatography coupled to tandem MS (HPLC-MS/MS) analysis [35]. These methodological advances are particularly beneficial in clinical settings, where speed, reproducibility, and minimal sample volume are essential.

DA, in particular, is increasingly studied not only for its role in neurological disorders but also as a potential metabolic biomarker in inflammatory and autoimmune diseases such as psoriasis and psoriatic arthritis. Looby et al. demonstrated the utility of SPME coupled with high-resolution MS for serum metabolomic fingerprinting, highlighting DA and eicosanoids as relevant small-molecule targets [36]. This exemplifies the growing trend of applying green and miniaturized extraction techniques in untargeted or semi-targeted metabolomic workflows. E has also been successfully analyzed using alkyl polyglucoside-based SUPRAS in liquid-phase microextraction (LPME), representing an emerging green alternative to conventional organic solvents in bioanalytical sample preparation [21].

Another class of biologically significant compounds that are often overlooked in classical endocrine frameworks are the eicosanoids. These are locally acting lipid-derived hormones synthesized from arachidonic acid through cyclooxygenase, lipoxygenase, or cytochrome P450 pathways. Unlike traditional hormones that travel through the bloodstream to distant target organs, eicosanoids exert their effects primarily via autocrine or paracrine mechanisms. Their functions span a broad spectrum, including inflammation, immune modulation, vascular tone regulation, and platelet aggregation. Among them, PGs constitute a major subclass and have been widely studied for their role in acute and chronic inflammatory conditions. Looby et al. applied SPME [36] and in vivo SPME [19] to successfully extract and monitor PGs and other eicosanoids from serum and tissue samples. These studies illustrate the capacity of microextraction platforms to preserve labile lipid mediators and facilitate minimally invasive sampling.

Despite their diverse chemical structures (catecholamines being highly polar and hydrophilic, and eicosanoids being moderately lipophilic) the common analytical hurdles for both classes include their instability, susceptibility to enzymatic degradation, and the need for rapid, selective, and sensitive detection. Microextraction techniques, particularly those based on sorbent- or solvent-assisted miniaturized formats, have proven instrumental in overcoming these challenges by enhancing analyte stability and facilitating clean-up from complex biofluids. These strategies align well with GAC principles and are increasingly favored in clinical, pharmacological, and translational research settings.

In summary, catecholamines, their metabolites, and eicosanoids such as PGs represent a functionally diverse and analytically demanding group of hormones. Their inclusion in bioanalytical studies is essential for a holistic understanding of neuroendocrine and inflammatory networks. Continued innovation in green microextraction technologies is likely to further enhance their routine analysis and biomarker utility.

## 3. Microextraction Techniques for Hormone Determination

Microextraction techniques have become central to contemporary bioanalytical workflows for hormones extraction since they address three concurrent needs: the analytical requirement to detect very low concentrations of diverse hormone classes in complex biological matrices; the pressure to reduce solvent use and waste in line with green analytical chemistry; and the practical demand for higher throughput and compatibility with modern hyphenated instrumentation. These techniques encompass a family of equilibrium- and partition-based approaches that minimize sample volumes and solvent consumption while providing preconcentration and some degree of matrix clean-up benefits that are especially valuable when dealing with trace-level hormones and scarce clinical samples of urine (Table 1) and other bioanalytical matrices (Table 2).

**Figure 3:**
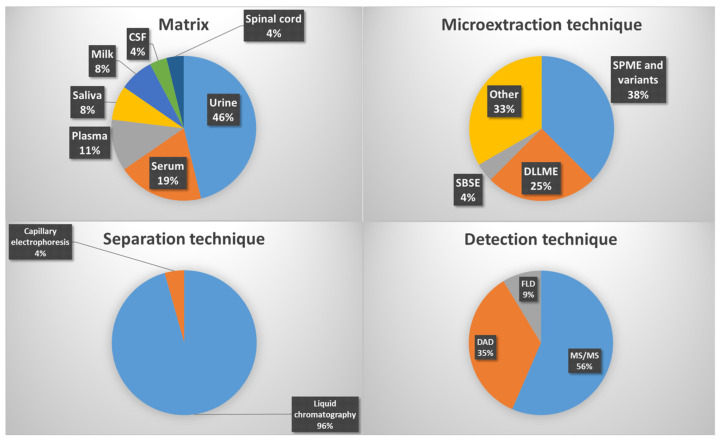
Relative percentages of the most common matrices, microextraction, separation and detection techniques regarding hormone studies.

Among all the studies summarized in Table 1 and Table 2, SPME and its variants are the most widely used (38%), followed by DLLME (25%), SBSE (4%), and other microextraction techniques (33%) (Figure 3). The most frequently analyzed matrices are urine (46%), serum (19%), plasma (11%), saliva and milk (8%), and cerebrospinal fluid (CSF) (4%). Regarding separation techniques, liquid chromatography is by far the most employed (96%), while for detection and quantification, MS/MS (56%), DAD (35%), and fluorescence detection (FLD) (9%) predominate.

### 3.1. Solid-Phase Microextraction and Variants

SPME is a widely adopted green sample-preparation technique that integrates extraction, concentration, and introduction into a single solvent-free process. It uses a coated fiber, blade, or in-tube device to adsorb analytes directly from biological matrices, followed by desorption into chromatographic systems, such as LC [54] or GC [55], often coupled to headspace strategy [56,57,58,59]. Ideal for trace-level hormone determination, SPME offers high enrichment efficiency, minimal solvent use, and compatibility with automated platforms, making it well-suited for routine and high-throughput bioanalytical applications [60].

**Figure 4:**
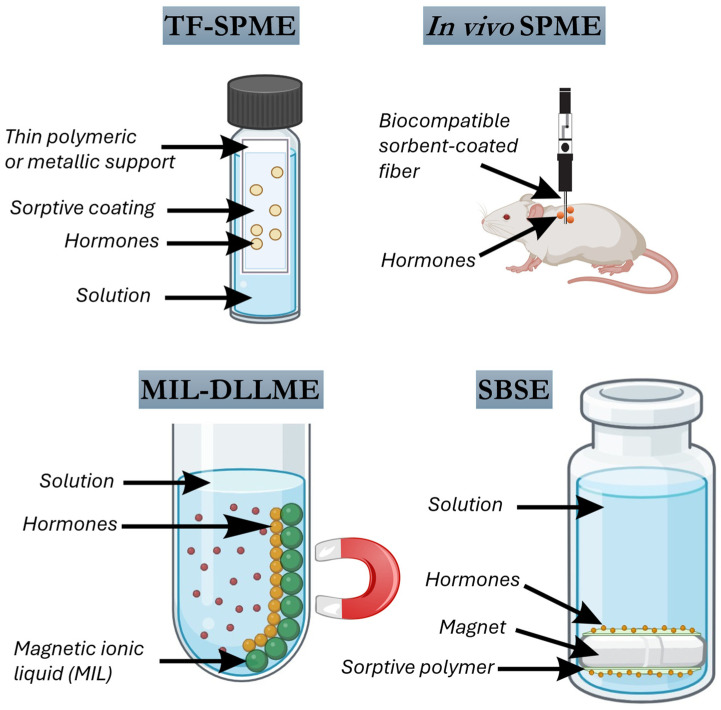
Main microextraction techniques for hormone extraction in bioanalytical matrices (some elements created with BioRender.com).

Some SPME variants have emerged recently. Thin-film solid phase microextraction (TF-SPME) represents a significant advancement over conventional fiber-based SPME, offering increased extraction efficiency, robustness, and compatibility with high-throughput bioanalytical workflows. Unlike classical SPME fibers, which are limited by small sorptive surface areas, TF-SPME devices utilize a planar geometry-typically a thin polymeric or metallic support coated with a sorptive phase-that provides a substantially larger surface area-to-volume ratio (Figure 4). This geometric advantage translates into improved sensitivity and faster extraction kinetics, features particularly desirable in the determination of trace-level hormones in complex biological matrices.

From a technical perspective, TF-SPME operates under the same general principles as traditional SPME, relying on the partitioning of analytes between the sample matrix and a sorptive coating [24]. However, the increased phase volume and surface area of thin-film coatings allow for enhanced analyte uptake without compromising extraction equilibrium. Various coatings have been explored in TF-SPME, including polydimethylsiloxane/divinylbenzene (PDMS/DVB) and mixed-mode phases tailored to improve selectivity [25]. From a GAC perspective, TF-SPME aligns with several key principles. The technique is inherently solvent-free, miniaturized, and amenable to automation, thus reducing reagent consumption, waste generation, and analyst exposure to hazardous solvents [24]. Moreover, TF-SPME enables direct sampling or extraction from small biological volumes, minimizing ethical and logistical burdens in clinical and ecological studies. Its reusability and potential for integration into online or in vivo platforms further enhance its sustainability profile. In summary, TF-SPME combines technical robustness, analytical sensitivity, and environmental compatibility. As this variant of SPME continues to evolve, future research is expected to focus on the development of novel sorbent materials with enhanced selectivity, integration with miniaturized or wearable devices, and standardization for clinical diagnostics.

In vivo SPME is a powerful sampling strategy that enables the direct, minimally invasive extraction of analytes from living systems without requiring sample collection, preparation, or destruction of the biological matrix. By inserting biocompatible, sorbent-coated fibers or probes directly into tissues, organs, or fluids, in vivo SPME allows real-time or near real-time monitoring of dynamic biochemical processes under physiological or experimental conditions (Figure 4). This approach is particularly valuable for tracking low-abundance endogenous compounds such as hormones, which are often subject to rapid metabolism, transport, or degradation. Therefore, in vivo SPME enables non-destructive, real-time sampling of living systems. Its convergence with advanced sorbent chemistries and high-resolution analytical instrumentation positions it as a transformative tool for dynamic endocrine monitoring in clinical and veterinary sciences.

Technically, in vivo SPME probes are typically coated with polymeric or nanostructured sorbent phases that exhibit selective affinity for target analytes, while maintaining biocompatibility and mechanical stability. The integration with LC-high resolution mass spectrometry (HRMS) provided a high-resolution, untargeted analytical platform, showcasing the potential of in vivo SPME in real-time endocrine profiling. From a GAC perspective, in vivo SPME offers several environmental and ethical advantages. The technique eliminates the need for extensive sample preparation, reduces solvent consumption to near zero, and minimizes biofluid volumes, thus aligning with the principles of waste reduction, energy efficiency, and miniaturization [61]. Moreover, the non-lethal, minimally disruptive nature of in vivo SPME facilitates longitudinal studies in both animal and plant systems, reducing the number of required specimens and enhancing experimental reproducibility [62]. In vivo SPME extends conventional SPME into living systems-minimally invasive fibers or swabs are directly inserted into tissues or fluids for real-time or repeated monitoring of endogenous compounds [19]. Future work will likely focus on the development of hormone-specific coatings, integration with wearable sensors, and clinical translation of this minimally invasive technology [63].

An innovative approach, in-tube SPME, automates extraction by routing the sample through a capillary coated internally with sorbent [29]. Emerging SPME variants include blade coatings enhanced with novel sorbents, such as covalent organic framework (COF) [47,49]. The COF’s inherent porosity and tunable chemistry make it an attractive platform for hormone enrichment. Additionally, SPME has made inroads in clinical metabolomic profiling [36].

### 3.2. Dispersive Liquid–Liquid Microextraction

DLLME has emerged as a powerful and versatile sample preparation strategy in bioanalytical chemistry, offering high enrichment factors, low solvent consumption, and rapid extraction kinetics. In its classical configuration, DLLME involves the rapid injection of a mixture of a dispersive solvent and a small volume of an extraction solvent into an aqueous sample, resulting in the formation of a cloudy solution due to fine dispersion of the extractant phase (Figure 4). This dispersion dramatically increases the contact surface area between phases, promoting rapid analyte partitioning and facilitating efficient preconcentration. A wide variety of hormone classes, including steroidal, thyroid, peptide, and neurotransmitter-related hormones, have been successfully extracted using DLLME and its numerous variants, tailored to meet the physicochemical properties of the analytes and the complexity of biological matrices.

One significant advancement is the use of ionic liquids and magnetic ionic liquids (MILs) as extraction solvents in DLLME, offering improved selectivity, tunable polarity, and enhanced greenness [53]. These MIL-based approaches eliminate the need for centrifugation or filtration steps due to their magnetic recoverability, streamlining the workflow and enhancing automation potential [45].

The DLLME with solidification of floating organic drop (DLLME-SFO) variant addresses environmental and operational concerns by employing low-density organic solvents with melting points close to room temperature (such as 1-dodecanol), which solidify upon cooling [64]. This technique has great potential for bioanalytical matrices with similar complexity, offering a more sustainable alternative to halogenated solvents.

Innovations in dispersive solvent selection and design have also expanded DLLME’s scope. DESs and NADESs are increasingly recognized for their biocompatibility and negligible volatility, making them ideal candidates for hormone determination in sensitive biological matrices. The integration of DESs aligns DLLME with the principles of GAC, promoting sustainability without compromising analytical performance, also in combination with molecularly imprinted solid-phase extraction (MISPE) [48].

The versatility of DLLME is further exemplified by its combination with hydrophilic interaction chromatography (HILIC) and ultrasound-assisted extraction [17]. Ultrasound enhances mass transfer and droplet dispersion, reducing extraction times and improving efficiency. Several adaptations of DLLME have been applied to steroidal hormone determination, such as MIL-DLLME [39,40]. Vortex-assisted DLLME represents another simple yet effective enhancement [51]. This vortexing approach eliminates the need for equipment like ultrasound baths, favoring miniaturized, portable setups. Effervescent-assisted LPME technique generates CO_2_ bubbles in situ, enhancing solvent dispersion and extraction efficiency, and represents a promising direction for non-invasive hormone monitoring [50].

DLLME has also shown compatibility with emerging device-based and lab-on-thread platforms, illustrating its potential in point-of-care diagnostics [65]. These devices significantly reduce solvent and sample volumes, enabling decentralized hormone monitoring. Some studies have integrated DLLME with microporous membrane-based LLE systems [41]. These configurations are suitable for routine screening and population-scale studies. Other forms of LPME such as SUPRAS-based extraction, for example, with hexafluoroisopropanol (HFIP), have also gained traction [21,37]. Hormone determination using DLLME and related strategies continues to evolve with the integration of chemometric tools, automated platforms, and advanced sorbent materials [34,43].

In summary, DLLME and its numerous variants-particularly those employing magnetic solvents, green extractants (DES, SUPRAS), and assisted dispersion techniques (ultrasound, vortex, effervescence) constitute a highly adaptable, sustainable, and effective set of tools for hormone determination in complex biological matrices. Their continual refinement and integration with emerging technologies position DLLME at the forefront of future bioanalytical workflows, especially in the context of GAC and decentralized health monitoring.

### 3.3. Stir Bar Sorptive Extraction

SBSE is a solventless microextraction technique that combines efficient analyte enrichment with operational simplicity, making it highly attractive for the determination of hormones in bioanalytical matrices [66]. SBSE involves the use of a magnetic stir bar coated with a sorptive polymer, typically PDMS, that is immersed in the liquid sample and agitated for a predetermined time (Figure 4). During this process, analytes partition from the sample into the PDMS coating according to their affinity for the hydrophobic phase. The volume of sorptive phase in SBSE (typically around 25 µL) is significantly larger than that of SPME (typically 0.5 µL), resulting in greater extraction capacity, higher preconcentration factors, and improved sensitivity. SBSE can be performed either in direct immersion mode or in the headspace configuration, where volatile compounds are extracted from the gas phase above the sample. After extraction, desorption of the retained analytes can be achieved by thermal desorption, usually followed by GC, or by solvent desorption, which is compatible with LC.

Although initially developed for the determination of volatile and semi-volatile nonpolar compounds, SBSE has evolved to include polar and even ionic analytes through the use of derivatization strategies or novel coatings beyond conventional PDMS. These include coatings with polar or functionalized polymers, as well as hybrid or composite phases with magnetic or nanostructured materials that improve selectivity and analyte-phase interactions. In the bioanalytical context, SBSE has demonstrated outstanding performance in the determination of steroid hormones in complex biological matrices such as urine, plasma, and serum, where matrix effects often compromise sensitivity and reproducibility with other extraction techniques [27].

The analytical benefits of SBSE-high enrichment factors, minimal solvent use, and robustness-make it especially suitable for trace-level hormone determination in bioanalytical applications. Its compatibility with diverse chromatographic systems (HPLC-MS and GC-MS) allows for broad applicability depending on the physicochemical properties of the target hormones. Moreover, SBSE’s reusability and cost-effectiveness are aligned with the principles of GAC. Despite these advantages, challenges remain regarding the selectivity of commercial coatings for more polar hormones and the limited availability of tailor-made stir bars. Recent developments aim to overcome these issues through the functionalization of stir bar surfaces with selective ligands or smart materials such as MIPs, potentially enhancing the scope of SBSE for protein-bound or hydrophilic analytes. As microextraction strategies continue to evolve, SBSE remains a valuable and adaptable technique for the sensitive, green, and efficient quantification of hormonally active compounds in complex biological matrices.

### 3.4. Other Emerging Microextraction Techniques

Emerging formats such as SBME, electromembrane extraction (EME), and single-drop microextraction (SDME), are gaining traction for miniaturized and automated hormone determination. SBME employs a small hollow fiber or polymeric tube-filled with a tailored organic or DES bar-immersed directly in the aqueous sample, allowing analyte diffusion into the solvent phase under stirring to accelerate mass transfer [67]. Following extraction, the bar is removed and the solvent analyzed by GC, LC or MS. Compared to SBSE, SBME uses a liquid phase which can be tuned to target non-polar to moderate polar steroid hormones, offering simplicity, low cost, and minimal solvent consumption [20].

SDME employs just a microliter-scale drop of organic solvent suspended in or immersed into the aqueous sample, effecting LLE in an extremely miniaturized, solvent-saving format. Originally introduced in the 1990s, SDME is green, inexpensive, and simple, though delicate to handle manually. While SDME has not yet been widely applied to hormone determination specifically, its principles-low solvent use, potential for automation, and high enrichment factors-make it promising for hormones in biofluids once adapted to high-throughput LC-MS workflows [68].

EME leverages an electrical field across a supported liquid membrane within a porous hollow fiber, driving electrokinetic migration of ionic and neutral analytes from aqueous samples into a small acceptor solution [69].

Dispersive pipette-tip micro SPE is a green, miniaturized technique ideal for extracting hormones from complex biological matrices. It uses a small amount of sorbent in a pipette tip, enabling efficient analyte retention through repeated aspiration-dispensing cycles. The method is simple, solvent-minimizing, and highly adaptable to various sorbents, allowing selective and sensitive hormone detection [52]. Its low cost, speed, and compatibility with automation make it well-suited for routine bioanalytical applications.

Together, these emerging techniques offer complementary capabilities. SBME achieves tailored liquid-phase extraction with green solvent design. SDME presents an ultra-miniaturized, solvent-saving drop format. EME enables electrically driven selective extraction of ionic hormones. Analytically, these methods can provide low LODs, high selectivity, low solvent use, and integration with chromatographic separation and MS detection. Future adaptation of SDME to hormone matrices and hybridization with automation and chemometric optimization could broaden their bioanalytical impact.

## 4. Green Solvents and Smart Materials in Hormone Microextraction

In recent years, the integration of green solvents and smart materials into microextraction methodologies has become a cornerstone of sustainable analytical chemistry. These innovations are driven by the principles of GAC, which emphasize minimizing the use of toxic reagents, reducing waste generation, and promoting safer, more energy-efficient analytical workflows [9]. Within this context, several solvent systems and material platforms have gained prominence due to their potential to replace conventional organic solvents and sorbents while maintaining or even enhancing analytical performance.

Among these advances, DESs and their natural analogs (NADESs) have attracted growing attention as designer solvents composed of hydrogen bond donors and acceptors that form eutectic mixtures with low melting points [48]. Their advantages include simple preparation, low volatility, tunable polarity, and biodegradability [23]. These properties make DESs particularly suitable for extracting both hydrophilic and hydrophobic hormones from complex biological matrices, often with improved selectivity and environmental compatibility [51].

Similarly, SUPRASs represent a new generation of self-assembled nanostructured liquids formed by the coacervation of amphiphilic molecules under controlled conditions [21]. Their unique microheterogeneous structure, consisting of dispersed nano-domains with tunable polarity and hydrogen-bonding capacity, allows selective solubilization of a broad range of analytes [37]. SUPRASs combine efficient extraction with low toxicity and reduced solvent consumption, aligning well with GAC principles.

In parallel, MIPs have emerged as highly selective sorbent materials designed through a template-based polymerization process that creates specific binding sites complementary to the target hormone molecule [48]. MIPs provide antibody-like recognition capabilities, exceptional stability, and reusability, offering a sustainable alternative to conventional immunosorbent-based approaches [70].

Finally, magnetic and nanostructured sorbents have significantly improved the efficiency and convenience of microextraction workflows. Magnetic nanoparticles enable rapid and simple phase separation using an external magnet, eliminating the need for centrifugation or filtration [52]. Moreover, MILs have recently emerged as a hybrid class of materials that combine the tunability and low volatility of ionic liquids with magnetic responsiveness, facilitating solvent recovery and automation of extraction processes [40]. These materials not only improve analytical performance but also contribute to greener and more sustainable extraction processes through reusability and reduced waste.

Together, these emerging solvents and smart materials represent a major step forward in developing environmentally friendly, selective, and efficient microextraction techniques for hormone determination in biological matrices.

### 4.1. Deep Eutectic Solvents

DES and their natural counterparts, NADES, have emerged as versatile and environmentally benign alternatives to conventional organic solvents for bioanalytical microextraction. These solvents are typically formed by combining a hydrogen bond donor with a hydrogen bond acceptor, resulting in a eutectic mixture whose melting point is significantly lower than that of its individual components. Their physicochemical tunability allows tailoring to the polarity and functional groups of specific hormone classes, thus enhancing extraction selectivity and efficiency. DES and NADES are especially advantageous in microextraction workflows such as DLLME, vortex-assisted liquid–liquid microextraction (VALLME), ultrasound-assisted LLE (UALLME), and SBME, offering low volatility, low toxicity, biodegradability, and strong hydrogen-bonding capability with analytes. They can be tailored for specific hormone classes and integrated into DLLME or LPME workflows.

Recent studies have demonstrated the analytical potential of non-ionic hydrophobic DES in bioanalytical applications. For instance, Guo et al. developed a strategy integrating MISPE with non-ionic hydrophobic DES-DLLME for the quantification of estrogens (E1, E2 and E3) in serum samples [48]. The DES phase, computationally optimized for minimal solvation energy and high surface interaction with estrogens, allowed for a remarkable 33–125-fold increase in sensitivity compared to conventional approaches. The enrichment was attributed to a charge similarity and strong dispersion interactions between the hydrophobic DES and the estrogenic compounds, providing both specificity and minimal matrix interference. Similarly, Zhao et al. reported the use of hydrophilic choline chloride-urea DES in combination with MNPs for extracting three sex hormones (E2, T, and P4) from various milk types [51]. Using VALLME coupled with magnetic solid-phase extraction (MSPE), this method achieved method detection limits (MDLs) as low as 1 ng mL^−1^ and recoveries exceeding 80%, highlighting the high affinity of DES for low-polarity hormone targets. The incorporation of DES also allowed effective pH control and eliminated the need for toxic solvents, positioning this methodology as a promising platform for hormone quantification in biological samples.

NADES, composed of natural, often biodegradable components such as organic acids, sugars, and amino acids, have demonstrated equally compelling properties for steroid hormone extraction. In a pioneering study by Andrade et al., a menthol–decanoic acid NADES was applied in ultrasound-assisted DLLME to extract E1, E2 and E3 from urine [23]. The extraction process was optimized using multivariate experimental design, and interaction mechanisms were further elucidated through molecular dynamics simulations. These simulations revealed that van der Waals interactions were the predominant force in analyte-solvent binding, a finding validated by the superior recoveries (82–98%) and reproducibility under optimized conditions. The study also demonstrated the utility of NADES in preserving analyte integrity while minimizing matrix effects, a critical advantage in hormonal determination of biological fluids. Complementing this, AL-Hashimi et al. designed an SBME approach using a hydrophobic NADES composed of menthol and lauric acid for the simultaneous extraction steroid hormones [20]. Computational predictions identified a 4:1 molar ratio as optimal for analyte-solvent interaction, confirmed experimentally by chemometric optimization. The system demonstrated excellent linearity (R^2^ ≥ 0.994), LODs below 0.4 µg L^−1^, and repeatability precision (RSD < 5%), highlighting its robustness and potential for clinical bioanalysis. Notably, the use of NADES confined within SBME devices provided a unique advantage by minimizing solvent consumption and enabling direct immersion into complex matrices without phase separation challenges.

Overall, DES and NADES represent a major advance in green sample preparation for hormone determination in bioanalytical matrices. Their ability to fine-tune solvation properties, combined with compatibility with modern microextraction formats, opens new opportunities for method development targeting a wide spectrum of hormone classes. Additionally, the integration of computational tools, such as molecular dynamics and chemometric optimization, allows for rational solvent design and performance prediction, further bridging GAC with precision bioanalysis.

### 4.2. Supramolecular Solvents

SUPRAS are structured nanostructured liquids formed through the spontaneous self-assembly of amphiphilic molecules in a given medium, typically water. These assemblies lead to the formation of well-organized supramolecular aggregates such as reverse micelles, vesicles, or liquid crystals, which serve as highly efficient and selective extraction phases for a broad range of analytes, including hormones. SUPRAS are structured liquids with amphiphilic properties that enable the extraction of a wide polarity range of hormones. Their tunability and high surface area enhance extraction efficiency [37]. Compared to conventional organic solvents, SUPRAS offer significant advantages in bioanalytical applications, including environmental compatibility, low toxicity, biodegradability, and the ability to tailor their composition for selective interactions with target analytes. While some characteristics of SUPRAS may intersect with those of other green solvents, such as DES, their self-assembly mechanisms and micellar architectures distinguish them both in formation and in analytical functionality.

Recent studies have demonstrated the potential of SUPRAS in LPME protocols for hormone determination in complex biological matrices. One particularly innovative development is the use of amphiphilic SUPRAS formed from 1,2-hexanediol and HFIP, which self-assemble into large spherical aggregates (10–100 µm) under aqueous conditions. These SUPRAS exhibit high stability across a wide pH range (2–12) and resist salinity effects, which is particularly relevant for biofluids such as urine and plasma. Li et al. developed and validated a SUPRAS-based LPME method using this system for the multiclass determination of ten World Anti-Doping Agency-listed anabolic hormones and metabolic modulators in human urine [37]. The method, coupled with HPLC-MS/MS, achieved high extraction efficiency (>80%), minimal matrix effects, and MDL as low as 0.018 ng mL^−1^, far below required performance levels. The high surface area and amphiphilicity of the SUPRAS allowed for fast analyte partitioning within 30 s, demonstrating the suitability of these solvents for time-sensitive and high-throughput applications in anti-doping and clinical bioanalysis.

A complementary approach involves carbohydrate-based amphiphilic systems. In a pioneering study, Vakh et al. reported for the first time the formation of SUPRAS using alkyl polyglucosides (caprylyl/capryl glucoside) in combination with medium-chain carboxylic acids as coacervating agents [21]. These systems exploit hydrogen bonding, hydrophobic interactions, and π-π stacking to generate structured liquids with sufficient hydrophobic domains to encapsulate small polar hormones. This strategy was applied to the extraction of E (a catecholamine hormone and adrenal biomarker) from human urine, yielding recoveries of up to 95%. Furthermore, the SUPRAS obtained displayed reduced viscosity and high compatibility with HPLC using FLD. The simplicity of extract recovery by decantation, due to phase rigidity at low temperature, added an operational advantage. These features position alkyl polyglucoside-based SUPRAS as effective, green alternatives to traditional surfactant-based systems, particularly for the preconcentration of polar hormones from aqueous biofluids.

Analytically, SUPRAS-based microextraction strategies show promise for their capacity to enrich target analytes efficiently while minimizing matrix effects and solvent use. Their adaptability to various detection systems, particularly HPLC coupled to MS/MS or FLD, broadens their scope for routine and regulatory applications. Moreover, their tunability through the choice of amphiphile, coacervating agent, and additive molecules enables fine control over selectivity and solvent polarity, which is critical when working with structurally diverse hormones ranging from polar catecholamines to non-polar steroidal hormones. Although their integration into in vivo or in situ sampling techniques remains limited, ongoing developments in SUPRAS formulation and stabilization suggest promising directions for real-time hormone monitoring. In the broader context of microextraction methods, SUPRAS represent a chemically intelligent and green platform that aligns well with the principles of miniaturization, sustainability, and analytical precision in bioanalytical hormone determination.

### 4.3. Molecularly Imprinted Polymers

MIPs have emerged as powerful recognition elements in bioanalytical microextraction due to their outstanding molecular selectivity, thermal and chemical stability, and compatibility with various sample preparation formats. MIPs are synthetic polymers engineered with template-specific binding sites that mimic natural receptor–analyte interactions, enabling them to selectively rebind target molecules even in the presence of structurally similar interferents. This unique property makes them particularly valuable in the selective enrichment of hormones from complex biological matrices, where low concentrations and high background noise present significant analytical challenges. When integrated with microextraction strategies, such as SPME, dispersive SPME, and MSPE, MIPs enhance the overall selectivity, reduce matrix interferences, and significantly improve signal-to-noise ratios [48].

One of the most promising aspects of MIP-based microextraction is its adaptability to specific hormone classes. For instance, estrogenic compounds such as E1 and E2, which exhibit structural similarities and occur at low concentrations in biological samples, have been efficiently preconcentrated using MIP-based protocols. Guo et al. developed a novel platform combining MIP-SPE with non-ionic hydrophobic DES-DLLME, demonstrating remarkable sensitivity enhancement for estrogens in serum [48]. The hybrid approach leveraged the template-specific binding of MIPs alongside the high partitioning capacity of DESs, yielding detection improvements, while effectively suppressing matrix effects. These results highlight the synergistic effect of combining green solvents with molecularly imprinted sorbents for trace-level hormone detection in bioanalytical contexts.

MIPs are typically synthesized through bulk, precipitation, suspension, or surface imprinting polymerization, each method offering different control over particle size, porosity, and binding site accessibility. Surface imprinting has gained particular traction in microextraction applications, as it allows faster mass transfer and more accessible binding sites, thereby improving kinetics and reusability. Furthermore, the use of MNPs as core supports for MIPs has facilitated magnetic dispersive SPE, enabling rapid and solvent-free separation of the sorbent from complex biofluids like plasma and urine. For example, magnetic MIPs synthesized for the recognition of E2 have shown excellent recoveries (>98%) and low LODs [70]. Such results not only underline the sensitivity and robustness of MIP-based techniques, but also support their scalability for routine hormone monitoring.

Moreover, recent trends emphasize the importance of greener MIP synthesis approaches, including the use of biodegradable monomers, ionic liquids, and DESs as polymerization media or dispersive agents. These innovations not only align with GAC principles but also improve analytical performance by reducing background contamination and enhancing analyte recovery. In addition, integrating MIPs with miniaturized formats such as in-tube SPME or microfluidic devices offers great potential for automation, high-throughput analysis, and clinical point-of-care testing. Despite some limitations in template design and polymerization reproducibility, MIPs represent a versatile and evolving class of smart materials that significantly enhance the analytical capabilities of microextraction strategies for hormone determination in complex biological systems.

### 4.4. Magnetic and Nanostructured Sorbents

Magnetic and nanostructured sorbents represent a powerful class of materials that have significantly enhanced the performance of microextraction techniques in bioanalytical applications, particularly in hormone determination. Their distinct physicochemical characteristics-such as high surface-area-to-volume ratio, tunable surface chemistry, and superparamagnetic behavior-enable efficient extraction, rapid phase separation, and improved sensitivity, all while aligning with GAC principles by reducing solvent consumption and sample handling complexity. Among them, MILs have attracted growing attention due to their dual functionality, since they act simultaneously as extractant and magnetically responsive phase. For example, Will et al. developed a DLLME method using MILs for multiclass analyte determination in urine, including the hormone E1 [40]. The optimized procedure showed LODs down to 3 µg L^−1^ and good repeatability, proving MIL-DLLME to be a feasible green alternative for hormone quantification.

A major advancement in this area is the use of phosphonium-based MILs, which provide visual recognition, high paramagnetism, and excellent extraction capabilities. Fan et al. combined ultrasound-assisted MIL-DLLME with ultra HPLC (UHPLC) coupled to triple quadrupole (QqQ)-MS^2^ to extract and quantify 20 neurotransmitters, such as E and NE, from rat spinal cord tissue [53]. The methodology holds strong relevance for hormone determination due to its low LODs, short extraction times, and broad linear range. Similarly, Ding et al. demonstrated the successful application of MIL-based liquid–liquid microextraction (MIL-LLME) for simultaneous extraction of the same NTs in human CSF and plasma, achieving high selectivity without the need for centrifugation [45]. This work highlights the method’s applicability to trace-level analysis in clinically relevant matrices and its potential to be translated to steroid and peptide hormone determination.

In parallel, magnetic nanocomposites have proven to be highly effective solid sorbents due to their ease of functionalization and enhanced extraction kinetics. A notable example is the magnetic-assisted dispersive pipette-tip micro SPE strategy introduced by Manouchehri et al., where a neodymium-based oscillating magnetic field was employed to activate a novel nanocube sorbent [52]. This platform enabled efficient enrichment of four synthetic steroid hormones in breast milk, with LODs below 0.02 ng mL^−1^ and recoveries above 85%. The magnetic actuation eliminated the need for centrifugation or vortexing, offering a fast and highly reproducible method suitable for automation.

Nanostructured materials are often tailored with MIPs, graphene derivatives, or cyclodextrins to enhance selectivity toward specific hormone targets. Functionalizing MNPs with MIPs creates hybrid sorbents with high molecular recognition, enabling selective preconcentration of structurally similar hormones in complex matrices. Similarly, cyclodextrin-modified magnetic nanomaterials can encapsulate hormone molecules through host-guest interactions, significantly improving extraction specificity. These surface modifications align with GAC goals by minimizing sample preparation steps while ensuring reliable quantification.

Overall, magnetic and nanostructured sorbents are increasingly recognized as enabling technologies for bioanalytical microextraction. Their high sorption capacities, rapid magnetic separation, and compatibility with a range of instrumental techniques make them ideal candidates for next-generation hormone assays. Additionally, their miniaturized format and adaptability to automation platforms support broader implementation in clinical and pharmacokinetic studies, while contributing to sustainability through reduced reagent consumption and waste generation.

## 5. Application of Microextraction Techniques in Bioanalytical Matrices

**Figure 5:**
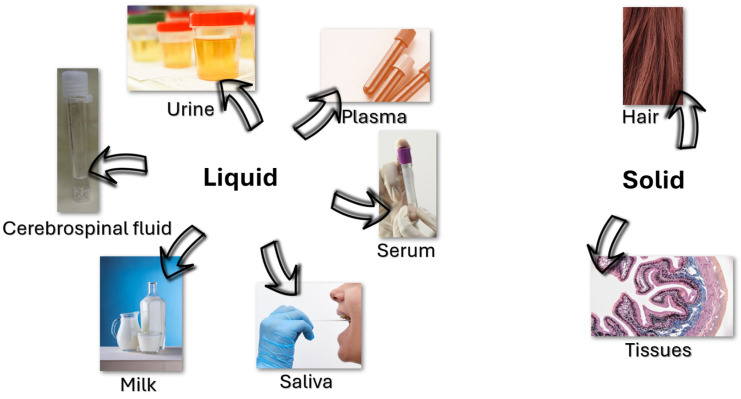
Most common bioanalytical matrices containing hormones.

The effectiveness of microextraction methods for hormone determination depends largely on the characteristics of the biological matrix. Bioanalytical samples differ widely in composition, complexity, and hormone distribution, which can affect extraction performance, analyte stability and analytical performance (Table 1 and Table 2). The most common matrices may include urine, plasma, serum, saliva, milk, CSF, hair, and tissues. These can be broadly grouped into fluid matrices (urine, plasma, serum, saliva, milk, CSF) and solid or semi-solid matrices (hair, tissues), each requiring tailored strategies for optimal extraction (Figure 5).

Urine is widely used due to its non-invasive collection and large volumes, but its variable dilution and pH can complicate extraction. Plasma and serum, rich in circulating hormones, present challenges due to high protein content and potential matrix effects. Saliva offers a simple, non-invasive alternative for free hormone measurement, though it typically contains low hormone levels and is prone to enzymatic degradation. Milk, with its complex lipid content, requires additional steps for phase separation and lipid removal. CSF is a clean, protein-poor matrix useful for assessing CNS hormones, but its collection is invasive and volume-limited. Hair provides a long-term record of hormone exposure but demands effective decontamination and digestion. Tissue samples offer spatial information but are complex and prone to degradation.

Across all matrices, hormone stability is a critical concern. Many hormones degrade due to enzymatic activity, temperature shifts, or pH changes, underscoring the importance of proper sample handling and storage. As such, the compatibility of microextraction techniques with each matrix must consider not only the chemical nature of the hormone but also the physicochemical properties of the sample. The following sections examine how these techniques are applied to each matrix, highlighting recent developments and methodological considerations.

### 5.1. Extraction of Hormones in Urine

Urine is one of the most widely used non-invasive matrices for hormone monitoring due to its easy accessibility, low cost, and the fact that many hormones and their metabolites are excreted through this route. Unlike matrices such as plasma or serum, urine reflects the cumulative excretion of hormones and their metabolites over time, offering a broader temporal window for hormonal profiling. However, the complex composition of urine (variable pH, salt content, and interferences) and the typically low concentrations of analytes necessitate highly sensitive and selective extraction techniques to ensure reliable quantification. Microextraction techniques have emerged as powerful tools to address these challenges, enabling selective, sensitive, and environmentally friendly sample preparation strategies tailored to hormones physicochemical properties.

Steroidal hormones are among the most frequently analyzed in urine, given their clinical relevance in endocrinology, oncology, and anti-doping control. Techniques such as TF-SPME have demonstrated high efficiency in extracting a broad panel of steroidal compounds. For instance, Struck-Lewicka et al. developed and validated a TF-SPME method for the metabolomic profiling by HPLC-Q-time-of-flight (TOF)-MS/MS of eight steroid hormones (E2, T, 17-OHP, DHEA, DHT, P4, androsterone and pregnenolone) in urine [25]. The extraction process was optimized using two sorbent coatings: polystyrene-divinylbenzene (PS-DVB) and polyacrylonitrile C18, with PS-DVB offering superior extraction efficiency. Among various desorption solvents tested, a 1:1 (*v*/*v*) mixture of acetonitrile and methanol yielded the highest analyte signals. Moreover, enzymatic hydrolysis using β-glucuronidase was employed to deconjugate glucuronide- and sulfate-bound steroid metabolites, significantly enhancing signal intensities. Extraction efficiencies ranged from 79% to 99% and recoveries from 89% to 112%, being the most abundant hormones P4, 17-OHP, T and androsterone, while E2 could only be identified in some samples. Matrix effect of the present method was between 1.4 and 11%. Similarly, Rajska et al. applied TF-SPME coupled with HPLC-QqQ-MS/MS to quantify androgens in women with PCOS, revealing significantly elevated levels of T, androstenedione, and DHT in affected individuals [24]. This methodology had high linearity (0.1–100 ng mL^−1^) with R^2^ > 0.994, LODs ranging from 0.04 to 0.09 ng mL^−1^ and limits of quantification (LOQs) from 0.1 to 0.3 ng mL^−1^. Repeatability was found to be 86–115% with CV < 8%, while reproducibility was 87–109% with CV < 11%. Rajska et al. also evaluated the recovery (81–109%) and the matrix effect (<12%) of the present method.

The use of green solvents has also gained traction in urine analysis. NADES, composed of menthol and decanoic acid, have been successfully employed in UALLME for the determination of E1, E2 and E3. Andrade et al. combined molecular dynamics simulations with experimental optimization, extracting by UALLME the hormones E1, E2 and E3 from urine samples, with quantification by HPLC-diode array detection (DAD) [23]. Linearity was studied from 10 to 750 µg L^−1^, with R^2^ > 0.991. LODs ranged from 3 to 8 µg L^−1^, LOQs from 10 to 25 µg L^−1^, repeatability RSDs from 8 to 19% and accuracy from 82 to 98%.

Another study by AL-Hashimi et al. introduced a NADES SBME method, demonstrating high extraction efficiency (91–95%) for steroid hormones (E2, T and P4) in urine, with quantification by HPLC-DAD [20]. Linearity was studied from 1.4 to 10,000 µg L^−1^ with R^2^ > 0.994. LODs were in the range of 0.278–0.407 µg L^−1^, while LOQs were 0.929–1.357 µg L^−1^. This method showed low repeatability (2.2–5.1%) and reproducibility (2.7–7.1%) RSDs.

SUPRAS have also shown promise in this matrix. Li et al. demonstrated the synthesis and application of SUPRAS, based on 1,2-hexanediol and hexafluoroisopropanol (HFIP), with HPLC-QqQ-MS/MS, achieving extraction efficiencies above 80% for six prohibited hormones (T, fluoxymesterona, oxandrolone, stanozolol, budesonida and ciclesonida) listed by the World Anti-Doping Agency [37]. This method presented high linearity (R^2^ > 0.990), with LODs from 0.018 to 0.18 ng mL^−1^, and LOQs from 0.06 to 0.6 ng mL^−1^. Repeatability was 88–120% with RSD < 19%, while reproducibility was 90–120% with RSD < 13%. Similarly, Vakh et al. also employed SUPRAS formed from alkyl polyglucosides, reinforcing their potential as eco-friendly alternatives to conventional solvents, for the extraction of E, achieving 95% recovery and compatibility with HPLC-FLD [21].

SPME and its variants continue to be widely applied. Zhang et al. developed a monolithic column SPME method for the quantification of six estrogens (E1, α-E2, β-E2, EE2, diethylstilbestrol and hexestrol) in urine by UHPLC-MS/MS, achieving recoveries between 76 and 107% with RSDs ranging from 2 to 8% [38]. Linear range from 0.1 to 25 µg L^−1^ had R^2^ > 0.995, with LODs comprised between 8.6 and 37 ng L^−1^ and LOQs between 28 and 100 ng L^−1^. RSDs for repeatability and reproducibility were 6.2–8.1% and 2.7–7.2%, respectively. In another study, Noori et al. developed and optimized an SBSE method using commercial PDMS-coated stir bars to isolate 17-OHP, obtaining recoveries between 87.5% and 101% in urine by HPLC-DAD [27]. Linearity studied from 2.4 to 2000 ng mL^−1^ had R^2^ > 0.996, with LOD of 0.8 ng mL^−1^. RSDs for repeatability and reproducibility of the method were 0.6–5% and 0.1–2.3%, respectively. The method’s green character was confirmed using multiple assessment tools, reinforcing SBSE’s compatibility with sustainable analytical practices.

DLLME has also been adapted for urinary hormone determination. Dmitrieva et al. employed DLLME combined with derivatization and UHPLC-QTOF-MS/MS to detect sixteen different ketosteroids, achieving recoveries from 79% to 98% [39]. Linearity was studied from 0.25 to 100 ng mL^−1^, with R^2^ > 0.996, while LODs were comprised between 0.1–0.25 ng mL^−1^ and LOQs between 0.25–1 ng mL^−1^. RSDs values for repeatability and reproducibility were 2.5–14.3% and 4.7–14.8%, respectively. Will et al. applied MILs in DLLME for the urine extraction of E1 and EE2 and quantification by HPLC-DAD, reporting recoveries from 97% to 119% for E1 and 87–103% for EE2 [40]. Linear range was studied from 10 to 250 µg L^−1^ with R^2^ > 0.992, LOD was 3 µg L^−1^ and LOQ was 10 µg L^−1^. Precision for repeatability and reproducibility was 6–18% and 14%, respectively. Zhou et al. used ultrasound-assisted IL-DLLME coupled with HILIC for the extraction of underivatized neurotransmitters in urine from dementia patients, demonstrating applicability to polar and unstable analytes [17].

Innovative materials have further enhanced extraction performance in urine samples. Turazzi et al. synthesized polyaniline-silica films doped with oxalic acid for TF-SPME, achieving recoveries between 71% and 115% for E1, E2, and 17-α-EE2 when quantified by HPLC-FLD [42]. Lopes et al. applied hollow fiber microporous membrane LLE within a 96-well plate format enhancing throughput while maintaining microextraction benefits. This method enabled determination of estrogens (E1, E2 and EE2) by HPLC-FLD with recoveries ranging from 82 to 118% [41]. Linearity was studied from 0.1 to 300 µg L^−1^ with R^2^ > 0.993, LODs were 0.03–15 µg L^−1^ and LOQs 1–50 µg L^−1^. Precision for repeatability and reproducibility was 1–13.3% and 7.3–18.1%, respectively.

Pieckowski et al. demonstrated the compatibility of DLLME with CE-DAD, using cyclodextrin-surfactant complexes for enhanced solubility and resolution, for the simultaneous determination of five steroid hormones (P4, T, epitestosterone, cortisol and corticosterone) in urine [43]. Linearity studied from 5 to 750 ng mL^−1^ presented R^2^ > 0.998, while LODs were 1.5–3 ng mL^−1^ and LOQs 5–10 ng mL^−1^. CV of the method was between 3.3 and 14.7%. Moreover, Pieckowski et al. further enhanced CE sensitivity for THs using electromigration techniques and laser-induced fluorescence, demonstrating applicability to real urine samples [32].

The relevance of urinary hormone determination extends beyond clinical diagnostics to environmental and toxicological studies. Rodriguez-Carrillo et al. linked urinary pesticide metabolites with altered reproductive hormone profiles in adolescents, highlighting the utility of urine in exposome research [34]. Peng et al. demonstrated associations between urinary pesticide biomarkers and THs levels in women, reinforcing the matrix’s value in endocrine disruption studies [31].

In summary, urine is a highly informative matrix for hormone determination, offering a non-invasive and integrative view of endocrine status. The application of green and emerging microextraction techniques-ranging from TF-SPME and DLLME to SUPRAS and NADES-based methods-has significantly improved the sensitivity, selectivity, and sustainability of hormone determination in urine. These advances not only enhance analytical performance but also align with the principles of GAC, paving the way for more ethical and efficient bioanalytical workflows.

### 5.2. Extraction of Hormones in Plasma

Plasma, the liquid component of blood devoid of cells but rich in proteins, electrolytes, and biomolecules, is a critical matrix in bioanalytical chemistry due to its direct reflection of systemic physiological and pathological states. Its relatively high protein content and complex composition pose unique challenges for hormone determination, particularly in terms of matrix effects, analyte stability, and binding interactions. Unlike matrices such as urine or saliva, where hormones are often present in free or conjugated forms, plasma contains both free and protein-bound hormone fractions, necessitating extraction techniques that can efficiently isolate target analytes without compromising their integrity or altering their distribution. Microextraction techniques have emerged as powerful tools to address these challenges, offering high selectivity, minimal solvent consumption, and compatibility with downstream analytical platforms.

SPME and its miniaturized variants have been widely applied for plasma hormone determination due to their ability to handle small sample volumes while maintaining high sensitivity. For instance, Skiba et al. developed a robust HPLC-MS/MS method using C18 MEPS for the quantification of leuprolide, a GnRH analog, in human plasma, achieving recoveries of 64% [44]. Linearity was studied from 0.05 to 40 ng mL^−1^ with R^2^ > 0.999, while the LOQ was 0.05 ng mL^−1^. Inter-run precision CV were between 3.6 and 8.0%, while intra-run precision CV were 5.2–7.2%. Authors demonstrated the absence of matrix effect, probably due to the use of internal standards. This highlights the suitability of microextraction for detecting low-abundance peptide hormones in complex matrices. Similarly, Cibotaru et al. proposed a novel approach combining microextraction and ultrafiltration to simultaneously determine free and total concentrations of T, along with plasma binding capacity, offering a more comprehensive understanding of hormone bioavailability in clinical samples [71]. This dual capability is particularly relevant in pharmacokinetics and personalized medicine, where the free hormone fraction often correlates more closely with biological activity.

MIL-LLME has also shown promise in plasma applications. Ding et al. demonstrated the use of MIL-LLME coupled with UHPLC-QqQ-MS/MS for the simultaneous determination of neurotransmitters in plasma and CSF, achieving high extraction efficiency and analytical precision [45]. Linearity was studied from 0.6 to 1200 µg L^−1^, although authors did not report R^2^ values. LODs were 0.20–0.27 µg L^−1^, while LOQs were 0.67–0.90 µg L^−1^. Recoveries were 96–107%, with RSDs values from 1.9 to 3.4%. RSDs for repeatability and reproducibility were 2.0–3.1% and 3.0–3.8%, respectively. Although the study focused on some neurotransmitters, such as E and NE hormones, the methodology is directly translatable to small, polar hormones such as catecholamines or thyroid ones. The use of magnetic solvents enables rapid phase separation without centrifugation, reducing processing time and enhancing throughput-an advantage in clinical and high-throughput settings.

In the context of steroid hormones, TF-SPME has been successfully applied to plasma samples. Maciążek-Jurczyk et al. developed a TF-SPME-HPLC-MS/MS method for the quantification of endogenous steroid hormones such as cortisol, T, and E2 in fish plasma, achieving low LODs and demonstrating high recovery and reproducibility [46]. Linearity was studied from 1 to 25 ng mL^−1^, with R^2^ > 0.998, while LODs and LOQs were 0.006–0.15 ng mL^−1^ and 0.02–0.50 ng mL^−1^, respectively. Recoveries were higher than 80%, accuracy RSDs lower than 6–15% and precision RSDs lower than 10–11%. Although the matrix differs from human plasma, the methodological principles and performance metrics underscore the potential of TF-SPME for hormone profiling in mammalian systems. The use of biocompatible coatings allowed direct extraction from plasma without prior cleanup, minimizing sample handling and preserving analyte integrity.

Plasma’s analytical relevance is further underscored by its central role in multiomics studies. Gladding et al. integrated SPME-based volatilomics with metabolomics and proteomics to investigate heart failure biomarkers in plasma, illustrating the matrix’s versatility for comprehensive molecular profiling [72]. Hormones, as key signaling molecules, are integral to such studies, and their accurate quantification in plasma is essential for elucidating disease mechanisms and therapeutic responses. Moreover, Lv et al. employed an HPLC-QTRAP-MS/MS method to quantify metabolites and lipids in human plasma, including some analytes such as steroid and peptide hormones, demonstrating the feasibility of large-scale hormone profiling with high analytical rigor [73].

In summary, plasma remains a gold-standard matrix for hormone determination due to its physiological relevance and accessibility. Microextraction techniques tailored to its unique characteristics-such as high protein content and analyte binding dynamics-enable sensitive, selective, and reproducible quantification of diverse hormone classes. Advances in sorbent materials, magnetic solvents, and integrated analytical platforms continue to expand the capabilities of microextraction in plasma, supporting its application in clinical diagnostics, pharmacokinetics, and systems biology.

### 5.3. Extraction of Hormones in Serum

Serum, the fluid component of blood obtained after coagulation and devoid of clotting factors, is a widely used matrix in clinical and bioanalytical studies due to its stability, reproducibility, and rich content of circulating biomarkers, including hormones. Compared to plasma, serum lacks fibrinogen and other clotting proteins, which can reduce matrix complexity and improve extraction efficiency for certain analytes. However, the high protein content and potential for hormone-protein binding still pose analytical challenges, particularly for low-abundance hormones such as estrogens, androgens, and peptide hormones. Microextraction techniques have proven to be highly effective in addressing these challenges, offering enhanced selectivity, reduced solvent consumption, and compatibility with sensitive detection platforms.

SPME has been extensively applied to serum samples for hormone determination. Zheng et al. applied a synthesized TpPa-1 COF via in situ layer-by-layer assembly directly onto an SPME fiber for the quantification by HPLC-MS/MS of sex hormones such as P4, T, and DHEA in human serum [47]. Linear range, studied from 0.1 to 100 ng mL^−1^, presented R^2^ > 0.994, with LODs of 0.023–0.75 ng mL^−1^ and LOQs of 0.07–2.5 ng mL^−1^. This method presented RSDs values for repeatability and reproducibility of 7.5–14.9% and 6.7–11.4%, respectively. Hormone concentrations found in women around 20 years old with PCOS, using this methodology, were 2.67–2.74 ng mL^−1^ (T), 1.01–1.07 ng mL^−1^ (P4) and 25.6–27.3 ng mL^−1^ (DHEA). The high surface area and tailored porosity of the COF coating enabled efficient extraction of structurally diverse steroid hormones, demonstrating the potential of advanced materials in serum microextraction. Similarly, Zhang et al. introduced a monolithic column-based SPME method coupled with UHPLC-MS/MS for the quantification of six estrogens (E1, β-E2, α-E2, EE2, diethylstilbestrol and hexestrol) in human serum [38]. This approach highlights the importance of optimizing sorbent chemistry and column architecture to enhance sensitivity and reduce matrix effects in serum analysis.

The integration of MIPs with microextraction has also shown promise in serum applications. Guo et al. employed non-ionic hydrophobic DESs in a DLLME setup in combination with MISPE to enrich estrogens (E1, E2, diethylstilbestrol and P4) in serum samples, significantly improving sensitivity when quantified by HPLC-DAD (up to 125-fold) and reducing interference from matrix components [48]. Linearity from 0.1 to 20 ng mL^−1^ presented R^2^ > 0.997, with LODs 0.08–0.4 ng mL^−1^ and LOQs 0.3–1.5 ng mL^−1^. RSDs values for repeatability and reproducibility were lower than 10.3%. The use of DESs not only aligns with GAC principles but also enhances analyte-solvent interactions due to their tunable polarity and low toxicity. This dual-extraction strategy exemplifies the synergy between selective sorbents and green solvents in complex matrices like serum.

In the context of endocrine disruption and environmental exposure, Rodriguez-Carrillo et al. investigated serum levels of kisspeptin, T, E2, LH, and FSH in adolescent males exposed to chemical mixtures, identifying kisspeptin as a potential biomarker of reproductive hormone dysregulation [34]. This study focusses on DLLME coupled to UHPLC-MS/MS, underscoring the analytical importance of serum for hormone biomonitoring and the need for sensitive, selective extraction methods to support such investigations.

SPME has also been employed in metabolomic studies involving serum. Looby et al. used SPME-HPLC-HRMS to profile serum metabolites in patients with psoriasis and psoriatic arthritis, detecting hormone-related metabolites and eicosanoids associated with disease activity [36]. Furthermore, Lv et al. applied an HPLC-QTRAP-MS/MS method to quantify over 144 analytes such as steroid and peptide hormones, validating the method with standard reference materials and applying it to clinical cohorts [73]. This large-scale approach illustrates the potential of serum-based microextraction for systems biology and precision medicine.

Although most studies focus on human serum, innovations in material science have also been tested in animal models. Yang et al. applied a sulfonic acid-functionalized COF coating for SPME blades for the extraction of monoamine neurotransmitters (E, NE and DA) in rat serum, before quantification by HPLC-FLD [49]. Linearity was studied from 0.1 to 300 ng mL^−1^, with R^2^ > 0.996, LODs of 0.015–0.03 ng mL^−1^ and LOQs of 0.05–0.1 ng mL^−1^. RSDs for repeatability and reproducibility were 2.6–6.6% and 5.9–8.5%, respectively. Recoveries for these hormones were in the range of 90–118%, with RSDs of 1.6–10.8%. Matrix effect was determined between 0.8% and 17.4%. These results suggest the potential applicability to hormone extraction in serum of this methodology, showing high capacity and chemical specificity, particularly for polar or charged species.

In conclusion, serum is a highly informative matrix for hormone determination, offering a balance between accessibility and analytical richness. Microextraction techniques-especially those incorporating advanced sorbents, green solvents, and hybrid strategies-enable sensitive and selective quantification of hormones in serum, supporting applications in clinical diagnostics, environmental health, and endocrine research. The continuous development of novel materials and integration with high-resolution analytical platforms will further enhance the capabilities of microextraction in serum-based hormone determination.

### 5.4. Extraction of Hormones in Saliva

Saliva is an increasingly valuable bioanalytical matrix for hormone determination due to its non-invasive collection, minimal sample volume requirements, and suitability for repeated or real-time monitoring in clinical and research settings. It reflects the unbound, biologically active fraction of hormones, providing insights into endocrine status without the interference of binding proteins typically present in blood-derived matrices. However, the low concentrations of hormones in saliva, along with the presence of mucins, enzymes, and variable pH, pose significant analytical challenges that require sensitive and selective microextraction strategies. These limitations have driven the development of tailored sample preparation approaches to enhance analyte recovery and minimize matrix effects, with an emphasis on green and miniaturized technologies.

Among hormones studied in saliva, steroidal compounds such as cortisol and T are of particular relevance due to their diagnostic value in stress-related disorders, endocrine dysfunctions, and metabolic syndromes. Dalanhol et al. introduced a novel effervescent-assisted LPME using a switchable hydrophilicity solvent system, achieving a simultaneous extraction of cortisol and T from oral fluid, demonstrating high precision and recovery rates, along with excellent greenness scores [50]. In this method, decanoic acid was used as a switchable solvent phase, activated in situ by acid–base reaction, and the extraction protocol required only 1 mL of saliva, thereby aligning with sustainability criteria and miniaturization principles. The optimized conditions allowed for effective extraction, with relative recoveries ranging from 99% to 105% for cortisol and 89% to 104% for T. Linear range of the HPLC-DAD method, from 15 to 750 ng mL^−1^, presented R^2^ > 0.992, while LOD and LOQ values were 4.55 ng mL^−1^ and 15 ng mL^−1^, respectively, which are adequate for physiological levels typically encountered in saliva. The method also demonstrated satisfactory repeatability (5.6–11.9%) and reproducibility (6.1–13.5%) precisions, highlighting its robustness for clinical applications. Khachornsakkul et al. coupled DLLME to a distance-based thread analytical device for melatonin detection in saliva, demonstrating the feasibility of integrating microextraction into paper-based or wearable analytical systems [65].

In parallel, online in-tube SPME coupled with HPLC-MS/MS has emerged as an effective automated non-invasive method for the determination of multiple steroid hormones in saliva, offering high throughput, sensitivity, and selectivity without the use of organic solvents [29]. This platform allowed for the simultaneous determination of nine steroid hormones (E1, E2, E3, cortisol, T, P4, DHEA, pregnenolone and aldosterone) within six minutes using a capillary column and positive ion multiple reaction monitoring. The system achieved low LODs (0.7–21 pg mL^−1^) with excellent linearity (R^2^ > 0.9990) and recovery values ranging from 82% to 114%, even with minimal sample preparation. Repeatability and reproducibility RSDs were lower than 7.5% and lower than 15%, respectively. Results from real samples showed that all E1, E2, E3 and some of pregnenolone (0.85–1.1 ng mL^−1^), P4 (0.10–2.5 ng mL^−1^), aldosterone (0.25 ng mL^−1^) and DHEA (0.43–0.83 ng mL^−1^) were below LOQ values for male patients, while cortisol (0.3–3.0 ng mL^−1^) and T (0.06–0.27 ng mL^−1^) were found in all of them. Regarding female patients, DHEA (0.5–1.1 ng mL^−1^), cortisol (0.3–4.8 ng mL^−1^) and E3 (0.7–1.1 ng mL^−1^) were present in all of them, while E2 (0.22–0.26 ng mL^−1^), pregnenolone (0.8–2.3 ng mL^−1^), P4 (0.1–3.6 ng mL^−1^) and T (0.05–0.12 ng mL^−1^) were found in most of the patients and E1 (1.1 ng mL^−1^) and aldosterone (0.86 ng mL^−1^) only in one. Importantly, the direct injection of ultrafiltrated saliva enabled streamlined processing, reduced sample manipulation, hands-on time, solvent consumption, and minimized analyte loss, positioning in-tube SPME as a promising tool for non-invasive endocrine monitoring in both clinical and field-based scenarios.

The advantages of saliva as a matrix, particularly when paired with green microextraction techniques, are evident in the context of pediatric, geriatric, and psychiatric assessments, where blood sampling may be impractical or ethically constrained. Compared to plasma or serum, salivary analysis avoids the complexity of protein binding and matrix interference, although it requires more sensitive instrumentation due to lower hormone concentrations. The use of effervescent-assisted LPME and in-tube SPME exemplifies how strategic solvent selection, automation, and sorbent design can overcome these limitations while enhancing method sustainability and compliance with GAC principles. Together, these methods expand the analytical toolkit for hormone profiling in saliva and contribute to the development of more accessible, environmentally responsible diagnostic protocols.

### 5.5. Extraction of Hormones in Milk

Milk is a complex biological matrix rich in proteins, lipids, carbohydrates, minerals, and vitamins, which poses significant analytical challenges for the trace-level determination of hormones due to matrix interferences and partitioning behaviors. Its composition not only varies among species and individuals but also changes with lactation stage, diet, and health status, thereby affecting extraction efficiency and analytical reproducibility. The presence of endogenous and exogenous hormones in milk, particularly steroidal compounds such as estrogens, progestogens, and androgens, has raised health concerns due to their potential endocrine-disrupting effects, especially in vulnerable populations like infants and children. Therefore, the development of accurate, sensitive, and environmentally sustainable microextraction strategies is critical for reliable hormone determination in milk matrices.

Among the most promising approaches, the combination of VALLME and MSPE has been successfully applied for the determination of three sex hormones (E1, E2, and P4) in various types of milk, including cow, goat, and infant formula [51]. In this method, DESs choline chloride-based systems, were employed as green extractants due to their low toxicity and tunable polarity, enhancing extraction efficiency for hydrophobic hormone molecules. Multi-walled carbon nanotubes (MWCNTs) act as magnetic sorbents capable of retaining the DES phase through hydrophobic and π-π interactions, allowing efficient preconcentration and cleanup. Under optimized conditions, HPLC-DAD method LODs and LOQs values were 1–1.3 ng mL^−1^ and 2.5–4.5 ng mL^−1^, respectively, with recoveries between 80% and 116% and repeatability RSDs of 2–11%, confirming the method’s applicability for trace determination in complex milk samples. Calibration curve from 0.1 to 50 µg mL^−1^ presented R^2^ > 0.990. Zhao et al. also demonstrated method validation across five milk types, where E2 was the most abundant hormone (2.0–4.6 ng mL^−1^), followed by T (0.2–0.3 ng mL^−1^) and P4 (0.2 ng mL^−1^), reinforcing the robustness and versatility of this hybrid extraction approach [51].

Another innovative strategy involves the use of magnetic-assisted dispersive pipette-tip MSPE, integrating a novel reciprocating magnetic field device to enhance sorbent interaction in breast milk. In this study, a calcined graphene oxide/silica composite doped with Co-Fe nanocubes was synthesized as a high-capacity sorbent and packed into a modified pipette tip [52]. The reciprocating magnetic field ensured uniform dispersion and maximized contact surface, facilitating rapid extraction of synthetic hormones such as triamcinolone, drospirenone, cyproterone and medroxyprogesterone. Following elution and HPLC-ESI-MS/MS analysis, LODs of 0.01–0.02 ng mL^−1^ and LOQs of 0.03–0.05 ng mL^−1^ were achieved, with excellent linearity from 0.03 to 500 ng mL^−1^ (R^2^ > 0.996), recoveries from 34 to 74% and matrix effect of 80–85%, demonstrating suitability for pharmacological and food safety monitoring. According to Manouchehri et al., this method offers advantages in terms of miniaturization, speed, and solvent economy, addressing several key aspects of GAC [52].

Compared to other biological matrices such as urine or serum, milk poses specific challenges including high lipid content, natural emulsification, and protein binding of analytes, which complicate phase separation and reduce free hormone availability for extraction. Consequently, the choice of sorbents and solvents must account for these unique characteristics to ensure efficient analyte release and minimize matrix effects. In this context, nanostructured materials like MWCNTs and graphene-based composites have shown superior performance, due to their high surface area and tailored functionalization, enabling selective interaction with target hormones even in lipid-rich environments.

Beyond synthetic hormones, endogenous estrogens such as E1 and E2 have also been monitored in milk for physiological and regulatory purposes. Since these hormones can naturally occur in ruminant milk or be introduced through veterinary treatments, accurate quantification is essential to assess consumer exposure and ensure compliance with food safety standards. Emerging microextraction strategies thus play a pivotal role in routine surveillance, toxicological risk assessment, and pharmacokinetic studies related to lactation and drug transfer into milk.

In summary, recent advances in microextraction techniques have significantly improved the analytical capabilities for hormone determination in milk. The integration of green solvents, magnetic materials, and device-assisted miniaturized formats offers high sensitivity, selectivity, and compatibility with complex matrices. These developments align with current trends toward sustainable and high-throughput analytical methodologies, reinforcing the critical role of microextraction in bioanalytical and food safety applications involving milk matrices.

### 5.6. Extraction of Hormones in Cerebrospinal Fluid

CSF represents a particularly valuable biological matrix in hormone research due to its close physiological contact with the CNS, offering a direct reflection of neuroendocrine activity in both physiological and pathological states. Unlike more accessible matrices such as urine or plasma, CSF is typically obtained through invasive lumbar puncture procedures, resulting in limited sample volumes and stricter ethical and logistical constraints. These characteristics underscore the importance of highly efficient, selective, and minimally invasive sample preparation techniques, especially when targeting trace-level endogenous compounds such as hormones. CSF hormone profiling is crucial in the context of neurological disorders, hypothalamic-pituitary axis dysregulation, and intracranial neoplasms, where even subtle fluctuations in neurohormonal concentrations can have significant diagnostic or prognostic implications. The application of microextraction techniques to CSF analysis offers a strategic advantage, aligning with GAC principles while enabling enrichment and isolation of low-abundance targets within a clean yet analytically demanding matrix.

One notable example of this is the implementation of MIL-LLME, a technique that demonstrates exceptional potential for extracting analytes from CSF without the need for centrifugation or laborious preparation steps. In a recent study, Ding et al. proposed an environmentally friendly and highly effective MIL-LLME method followed by UHPLC-QqQ-MS/MS for the simultaneous determination of twenty neuroactive compounds (such as E and DA) in human CSF, achieving high sensitivity and operational simplicity through magnetic phase separation without centrifugation [45]. The study optimized key extraction parameters including the type and volume of MIL, vortex speed, ionic strength, and pH, demonstrating that the selected MIL provided superior extraction efficiency for some hormones such as DA and E.

Compared to other biofluids, CSF presents a cleaner baseline, often free from extensive protein and lipid interference. However, the ultralow concentrations of hormones and the strict volume limitations call for extraction methods that combine microscale operation with high preconcentration capacity. Techniques like MIL-LLME fulfill these requirements, offering rapid extraction kinetics, straightforward phase separation via magnetic responsiveness, and a green profile due to low solvent consumption and absence of non-environmentally friendly reagents. Importantly, the non-volatile and thermally stable nature of MILs also enhances method robustness during downstream analytical steps. While clinical routine adoption of microextraction for CSF hormone determination remains limited, the results from MIL-LLME applications suggest a clear path forward in the integration of such green strategies into neuroendocrine diagnostics. As the demand for precise and sustainable bioanalytical tools continues to grow, microextraction techniques in CSF are likely to gain momentum, especially in translational research focusing on neurohormonal biomarkers.

### 5.7. Extraction of Hormones in Hair

Hair has emerged as a highly informative biological matrix for retrospective hormone determination, offering unique advantages over conventional fluids such as urine, serum, or saliva due to its ability to reflect long-term endocrine status without being influenced by short-term fluctuations or circadian variability [30]. As a keratinized tissue with a slow and predictable growth rate (approximately 1–1.5 cm per month), hair allows for the chronological mapping of hormone exposure over weeks to months, depending on the length of the strand analyzed. Unlike urine or plasma, which capture hormone concentrations at the time of sampling and are highly susceptible to acute physiological or environmental perturbations, hair provides a temporally integrated measure of hormonal burden, making it especially valuable for assessing chronic stress, endocrine disruption, and long-term exposure to xenobiotics.

In the context of microextraction, hair presents both challenges and opportunities. Its solid, fibrous nature requires appropriate pre-treatment steps such as washing, pulverization, or digestion prior to extraction. Once homogenized, however, microextraction techniques offer an efficient and solvent-minimized approach to isolate steroidal, thyroid, and glucocorticoid hormones from the complex hair matrix. SPE and miniaturized sorptive-based techniques, such as SPME, have been adapted for this purpose, often in combination with MS detection for enhanced selectivity and sensitivity. These techniques require minimal sample amounts (10–20 mg of hair) and are compatible with GAC principles by reducing toxic solvent use.

Among the hormones most frequently analyzed in hair are glucocorticoids, particularly cortisol and cortisone, which serve as robust biomarkers of stress and hypothalamic–pituitary–adrenal axis activity. In a recent study involving 196 healthy women from urban China, cortisol, cortisone, tetrahydrocortisol, and tetrahydrocortisone concentrations were determined in hair using SPME and associated with environmental exposure to various pollutants [26]. The results revealed statistically significant relationships between multiple pesticides and altered glucocorticoid profiles, suggesting that chronic exposure to xenobiotics may disrupt adrenal hormone homeostasis. Specifically, pollutants such as bisphenol S, carbofuran, and trifluralin were found to correlate with multiple glucocorticoid levels and their molar ratios, reinforcing the use of hair as a non-invasive matrix for both hormonal and exposomic assessment.

Hair analysis has also been extended to THs, providing an alternative for evaluating long-term thyroid function and its disruption due to environmental chemicals. In a cross-sectional analysis of the same cohort of women, Peng et al. measured T4, T3, rT3, and T2 in hair samples and identified significant associations with organophosphate pesticides, pyrethroids, and azole fungicides [31]. For instance, diethyl phosphate and difenoconazole were negatively associated with T4 while showing positive correlations with T3 levels, indicating potential disruption of peripheral conversion pathways. These results support the idea that hair TH profiling can reflect both endogenous regulation and exogenous perturbation of thyroid function, a perspective that blood-based snapshots may fail to capture.

Furthermore, hair sampling has been applied in veterinary and animal production settings to assess hormonal status in livestock. In sows, Peric et al. monitored cortisol and DHEA concentrations throughout gestation and postpartum periods as indicators of allostatic load [30]. Their findings demonstrated significant temporal shifts in hormone ratios depending on housing systems and batch effects, highlighting how hair integrates hormonal response to environmental and physiological stressors over time. The use of radioimmunoassay following hair homogenization and extraction provided robust analytical outcomes and underscored the practicality of non-invasive sampling for longitudinal studies.

In sum, hair represents a matrix of exceptional value for hormone determination in both clinical and environmental health research. Its capacity to capture long-term endocrine activity sets it apart from traditional biofluids and makes it particularly suitable for exposure-effect assessments involving EDC. Microextraction techniques, particularly those that embrace GAC principles, offer powerful tools for efficiently isolating hormones from hair with minimal sample manipulation. While challenges remain in standardizing hair preparation and addressing potential external contamination, ongoing methodological advancements and increased regulatory interest point toward a growing role for hair-based hormone testing in bioanalytical science.

### 5.8. Extraction of Hormones in Tissues

The extraction of hormones from tissue samples presents unique analytical challenges compared to liquid bioanalytical matrices due to the solid, heterogeneous, and lipid-rich nature of biological tissues. The complexity of these matrices often leads to substantial matrix effects, including signal suppression and analyte degradation, which can compromise the accuracy and reproducibility of hormone quantification. In this context, microextraction techniques-particularly SPME and DLLME-have emerged as powerful tools for the minimally invasive, solvent-saving, and selective isolation of hormones directly from tissue. These techniques offer a significant advantage in preserving the native distribution and chemical integrity of target analytes, especially in in vivo and in situ applications. Recent studies have demonstrated the feasibility of using in vivo SPME to extract hormonally active compounds from solid tissues such as porcine lung, mouse liver, brain, and rat spinal cord, enabling real-time monitoring of biochemical dynamics under physiological or pathological conditions. For instance, in a study on prolonged normothermic ex vivo lung perfusion, in vivo SPME fibers were directly inserted into porcine lung tissue to extract transient, low-abundance lipid mediators such as neuroprostanes, which are hormonally active eicosanoids involved in inflammatory signaling pathways [19]. This minimally invasive approach enabled the detection of hormonally relevant pathways and metabolites without extensive tissue processing, demonstrating the suitability of in vivo SPME for time-resolved hormonal determination in solid organs. Similarly, in a comprehensive metabolomics and lipidomics study involving mouse liver tissue, a high-throughput LC-MS method quantified a panel of over 1000 endogenous molecules, including steroidal and peptide hormones. Among these analytes were several hormone classes, confirming the applicability of metabolome-wide profiling strategies to tissue-based hormonal studies [73]. While this study combined both targeted and untargeted approaches, the robust quantification of tissue-based hormones highlights the potential of liver as a matrix for endocrine biomarker discovery. Newer studies, such as Peinado et al., suggest that hormone microextraction strategies like DLLME could be instrumental in exposome research, by enabling trace-level detection of EDC and hormone alterations in relation to gene expression profiles [74].

Brain tissue has also been explored for in vivo hormonal determination using microextraction strategies. Yang et al. developed a COF-based SPME fiber functionalized with sulfonic acid groups, which exhibited excellent selectivity, mesoporosity, and stability for the enrichment of endogenous compounds in the mouse brain, such as NE. The COF-SO_3_H-coated fibers achieved sub-nanogram per milliliter LODs, high precision, and minimal matrix interference, confirming their suitability for complex tissue environments where hormonal regulation is tightly coupled with neurotransmitter activity [63]. In another relevant application, Fan et al. used ultrasound-assisted MIL-DLLME to extract and quantify 20 hormonally active neurotransmitters from rat spinal cord, including E, NE and DA [53]. The magnetic properties and hydrophilic character of the selected ionic liquid enabled efficient dispersion and recovery of target analytes with LOD and strong analytical reproducibility. Calibration curves were from 0.51 to 2360 µg g^−1^, depending on the hormone, with R^2^ > 0.995 in all cases, with LODs varying from 0.15 to 0.64 µg g^−1^ and LOQs from 0.5 to 2.1 µg g^−1^. Recoveries obtained went from 94 to 118%, with RSDs being 2.6–7.5%. Repeatability RSDs were 2.1–6.8%, while reproducibility RSDs values were 3.7–8.0%. This work illustrates how ionic liquid-based DLLME methods can be optimized for solid tissue matrices with high protein and lipid content, allowing selective extraction of hormone-like molecules involved in neuroregulation [53].

Santos et al. applied a DLLME-SFO strategy after a QuEChERS clean-up to quantify E2 in fish muscle, highlighting its potential for trace hormone detection with reduced organic solvent use [64]. Liu et al., reported a mesoporous carbon hollow sphere-based in vivo SPME probe combined with HPLC-MS/MS to continuously monitor plant hormones (abscisic acid (ABA), indole-3-acetic acid and gibberellin A_3_) in living Chinese aloe, achieving low limits of detection (LODs) and relative standard deviation (RSD) [62]. Zhang et al., used in vivo SPME to track jasmonic, salicylic and ABAs in spinach exposed to pharmaceuticals, linking hormone modulation to physiological stress without sample destruction [61]. A study on acidic plant hormones (jasmonic acid, ABA, salicylic acid, gibberellic acid, etc.) demonstrated EME combined with HPLC-MS/MS achieved high sensitivity and selectivity (R^2^ > 0.995), LOQs from 0.1 to 10 ng mL^−1^, recoveries of 34–50%, and superior clean-up compared to conventional LLE, with minimal solvent consumption [69].

Together, these examples demonstrate that tissue-based hormone determination through microextraction is not only feasible but also increasingly sophisticated, with applications ranging from endocrine monitoring in organ transplantation to biomarker discovery in neurological disorders. Compared to fluid matrices, tissue extraction demands a higher degree of method customization due to the need for efficient matrix penetration, reduced analyte degradation, and compatibility with downstream analytical techniques such as HPLC-MS/MS. However, innovations in sorbent design, device miniaturization, and coupling strategies enable more precise and sensitive detection of tissue-based hormones. As the field progresses, tissue microextraction may offer valuable insights into localized hormone distribution, intracrine and paracrine signaling mechanisms, and the spatial dynamics of endocrine regulation across different physiological systems.

## 6. Critical Comparison and Challenges

The growing implementation of microextraction techniques in hormone determination reflects their capacity to address the sensitivity, selectivity, and environmental concerns associated with conventional sample preparation methods. However, a critical comparison of their performance reveals that each technique offers distinct advantages and limitations, and no single method can be considered universally optimal. To reinforce the conclusions drawn, a qualitative assessment of the methodological robustness of the included studies was also considered. Particular attention was paid to the validation status of analytical methods, reported recovery and precision values, the representativeness of the studied biological matrices, and the environmental sustainability of the proposed microextraction approaches. These elements were critically evaluated during data interpretation to ensure that the synthesis presented reflects both the strength and limitations of the available evidence, even though no formal scoring system was applied.

An additional aspect to be considered is the potential influence of funding sources on the reliability of reported results. In this regard, it is noteworthy that all studies included in this review explicitly disclosed their funding sources or provided conflict-of-interest statements, which contributes to the transparency and credibility of the available evidence. Although a formal analysis of funding-related bias was not performed, the consistent reporting of financial support across the reviewed works suggests that this factor is unlikely to have significantly influenced the conclusions drawn in this review.

SPME and SBSE are widely favored for their potential for automation, minimal solvent use, and high reproducibility. Their fiber- or stir bar-based formats facilitate integration with autosamplers and online analytical platforms, which is particularly attractive for routine bioanalytical workflows. In contrast, DLLME stands out for its rapid extraction kinetics and high enrichment factors, especially in applications requiring quick turnaround and minimal sample manipulation. More recent developments in materials science have introduced MIPs and nanostructured sorbents, which significantly enhance selectivity by tailoring molecular recognition sites and increasing surface area. Despite these advancements, several technical and operational challenges persist across platforms.

SPME, in particular, continues to face hurdles in selective sorbent development. Designing coatings with high affinity toward structurally diverse hormones is complicated by the wide range of polarity, molecular weight, and functional groups these compounds exhibit. Functionalized COFs and MIPs have shown promise in this context, yet achieving a balance between molecular specificity and coating robustness remains difficult. Moreover, matrix complexity, especially in biological fluids, introduces significant interference risks. The abundance of proteins, lipids, and other macromolecules can foul sorbent coatings, compromising extraction efficiency and reproducibility. Strategies such as the use of disposable fiber formats or regular sorbent regeneration protocols have been proposed to mitigate these issues but are not yet standardized.

Another critical limitation arises in quantitative determination. Since microextraction techniques like SPME often operate in a non-exhaustive extraction mode, accurate quantitation requires careful calibration approaches. Matrix effects can skew results unless adequately corrected through matrix-matched calibration, standard addition methods, or the use of stable isotope-labeled internal standards. These methods increase complexity and cost, particularly in high-throughput or clinical settings. Sensitivity is another bottleneck, especially for low-abundance hormones present at picogram or nanogram per milliliter concentrations. Achieving the necessary LODs pushes the performance boundaries of MS systems, requiring additional preconcentration strategies and high-selectivity sorbents to meet stringent analytical targets.

Beyond the extraction format, the incorporation of green solvents has added another layer of complexity to the performance landscape. DES and their natural analogs, NADES, have emerged as environmentally friendly alternatives to conventional organic solvents in DLLME and related LPME methods. Their tunable polarity, low volatility, and biodegradability make them appealing for hormone determination, especially in studies seeking to reduce toxic waste. However, the high viscosity of many DES formulations can hinder mass transfer and reduce extraction kinetics, requiring additional optimization of dilution ratios or temperature control. Similarly, SUPRAS, formed by the self-assembly of amphiphilic molecules, offer unique microenvironments that enhance selectivity through multiple non-covalent interactions. SUPRAS-based microextraction has shown promise for extracting hormones with diverse physicochemical properties, but challenges remain in reproducibility, large-scale synthesis, and compatibility with existing instrumentation.

Smart sorbent materials also play a pivotal role in defining the analytical capabilities of microextraction systems. MIPs, designed to recognize specific molecular templates, provide high selectivity but often suffer from slow binding kinetics, batch-to-batch variability, and limited reusability. On the other hand, magnetic and nanostructured sorbents, such as graphene oxide composites or MOFs, offer large surface areas and easy recoverability via magnetic separation. These materials have improved extraction efficiency and reduced processing time, yet their synthesis and functionalization can be labor-intensive, costly, and inconsistent. Additionally, concerns about nanoparticle stability, aggregation, and potential cytotoxicity have limited their use in clinical or regulatory environments.

From a practical standpoint, the selection of a microextraction technique is often driven less by theoretical performance and more by operational constraints such as ease of use, cost of materials, compatibility with existing analytical platforms, and required sample volumes. Techniques like DLLME, while rapid and inexpensive, may involve the use of hazardous dispersive solvents, which limit their appeal in GAC contexts. Conversely, SBSE provides high sorptive capacity but suffers from limited commercial coating diversity and longer extraction times, which may hinder throughput. Additionally, advanced materials such as nanocomposites or magnetic sorbents often require specialized synthesis protocols or pretreatment steps, increasing the methodological burden on laboratories.

Despite significant innovation, a major challenge across all techniques remains the lack of standardized protocols for hormone microextraction. Method development is often application-specific, resulting in poor comparability between studies. Moreover, there is limited guidance on harmonized validation criteria, which impedes the broader regulatory acceptance of microextraction-based methods. Interlaboratory studies are scarce, and most validation efforts focus on analytical figures of merit without considering long-term robustness or real-world matrix variability. As a result, the translation of these techniques into clinical or regulatory environments remains slow. In this context, chemometric tools and artificial intelligence are increasingly being explored to optimize experimental parameters, predict analyte-sorbent interactions, and streamline method development [72]. These approaches can reduce trial-and-error experimentation and improve reproducibility, especially when dealing with multivariate optimization of complex systems. Establishing consensus protocols, promoting interlaboratory harmonization, and aligning method validation with international guidelines are essential to ensure reproducibility, scalability, and regulatory compliance. Only through such efforts can the full potential of microextraction techniques be realized in routine hormone determination.

## Figures and Tables

**Table 1:** Figures of merit for hormone microextraction and determination techniques applied to urine matrices.

Hormones	Microextraction Technique	Green Extraction Solvent or Smart Material	Extraction Efficiency (%)	Separation and Quantification	Linearity Range	R^2^	LOD	LOQ	RSD (Repeatability) (%)	RSD (Reproducibility) (%)	Ref.
Steroids	TF-SPME	-	79–99	HPLC-QTOF-MS/MS	-	-	-	-	-	-	[25]
Androgens	TF-SPME	-	81–109	HPLC-QqQ-MS/MS	0.1–100 ng mL^−1^	0.994	0.04–0.09 ng mL^−1^	0.1–0.3 ng mL^−1^	8	11	[24]
Estrogens	UALLME	Menthol: decanoic/octanoic acid (1:1, 1:2, 2:1)	-	HPLC-DAD	10–750 µg L^−1^	0.991	3–8 µg L^−1^	10–25 µg L^−1^	8–19	-	[23]
Steroids	SBME	Menthol and lauric acid (2:1, 3:1, 4:1, 5:1)	91–95	HPLC-DAD	1.4–10,000 µg L^−1^	0.994	0.278–0.407 µg L^−1^	0.929–1.357 µg L^−1^	2.2–5.1	2.7–7.1	[20]
Doping hormones	LPME	1,2-hexanediol:HFIP:water	-	-	-	0.990	0.018–0.18 ng mL^−1^	0.06–0.6 ng mL^−1^	19	13	[37]
Estrogens	SPME	-	76–107	UHPLC-MS/MS	0.1–25 µg L^−1^	0.995	8.6–37 ng L^−1^	28–100 ng L^−1^	6.2–8.1	2.7–7.2	[38]
17-OHP	SBSE	Greenness evaluation	87.5–101	HPLC-DAD	2.4–2000 ng mL^−1^	0.996	0.8 ng mL^−1^	-	0.6–5	0.1–2.3	[27]
Ketosteroids	DLLME	-	79–98	UHPLC-QTOF-MS/MS	0.25–100 ng mL^−1^	0.996	0.1–0.25 ng mL^−1^	0.25–1 ng mL^−1^	2.5–14.3	4.7–14.8	[39]
Estrogens	DLLME	MIL	97–119	HPLC-DAD	10–250 µg L^−1^	0.992	3 µg L^−1^	10 µg L^−1^	6–18	14	[40]
Estrogens	Microporous membrane LLE	-	-	-	0.1–300 µg L^−1^	0.993	0.03–15 µg L^−1^	1–50 µg L^−1^	1–13.3	7.3–18.1	[41]
Estrogens	TF-SPME	-	71–115	HPLC-FLD	-	-	-	-	-	-	[42]
Steroids	DLLME	-	-	CE-DAD	5–750 ng mL^−1^	0.998	1.5–3 ng mL^−1^	5–10 ng mL^−1^	-	-	[43]

17-OHP, 17-hydroxyprogesterone; CE, capillary electrophoresis; DAD, diode array detection; DLLME, dispersive liquid–liquid microextraction; HPLC, high-performance liquid chromatography; LOD, limit of detection; LOQ, limit of quantification; LPME, liquid-phase microextraction; MIL, magnetic ionic liquid, MS/MS, tandem mass spectrometry; TOF, time of flight; RSD, relative standard deviation; SBME, solvent bar microextraction; SBSE, stir bar sorptive extraction; SPME, solid-phase microextraction; TF, thin-film; UALLME, ultrasound-assisted liquid–liquid microextraction; UHPLC, ultra-high-performance liquid chromatography.

**Table 2:** Figures of merit for hormone microextraction and determination techniques applied to plasma, serum, saliva, milk, CSF and tissue matrices.

Bioanalytical Matrix	Hormones	Microextraction Technique	Green Extraction Solvent or Smart Material	Extraction Efficiency (%)	Separation and Quantification	Linearity Range	R^2^	LOD	LOQ	RSD (Repeatability) (%)	RSD (Reproducibility) (%)	Ref.
Plasma	Leuprolide	MEPS	-	64	HPLC-MS/MS	0.05–40 ng mL^−1^	0.999	-	0.05 ng mL^−1^	5.2–7.2	3.6–8.0	[44]
Plasma	E and NE	LLME	MIL	96–107	UHPLC-QqQ-MS/MS	0.6–1200 µg L^−1^	-	0.20–0.27 µg L^−1^	0.67–0.90 µg L^−1^	2.0–3.1	3.0–3.8	[45]
Plasma	Steroids	TF-SPME	-	>80	HPLC-MS/MS	1–25 ng mL^−1^	0.998	0.006–0.15 ng mL^−1^	0.02–0.50 ng mL^−1^	-	-	[46]
Serum	Sex hormones	SPME	-	-	HPLC-MS/MS	0.1–100 ng mL^−1^	0.994	0.023–0.75 ng mL^−1^	0.07–2.5 ng mL^−1^	7.5–14.9	6.7–11.4	[47]
Serum	Estrogens	SPME	-	-	UHPLC-MS/MS	-	-	-	-	-	-	[38]
Serum	Estrogens	DLLME	HFIP–lauryl ether/polyethylene glycol	-	HPLC-DAD	0.1–20 ng mL^−1^	0.997	0.08–0.4 ng mL^−1^	0.3–1.5 ng mL^−1^	<10%	<10%	[48]
Serum	Kisspeptin, T, E2, LH and FSH	DLLME	-	-	UHPLC-MS/MS	-	-	-	-	-	-	[34]
Serum	E, NE and DA	SPME blade	Greenness evaluation	90–118	HPLC-FLD	0.1–300 ng mL^−1^	0.996	0.015–0.03 ng mL^−1^	0.05–0.1 ng mL^−1^	2.6–6.6	5.9–8.5	[49]
Saliva	Cortisol and T	Effervescent-assisted LPME	Decanoic acid	99–105	HPLC-DAD	15–750 ng mL^−1^	0.992	4.55 ng mL^−1^	15 ng mL^−1^	5.6–11.9	6.1–13.5	[50]
Saliva	Steroids	In-tube SPME	-	82–114	HPLC-MS/MS	-	0.9990	0.7–21 pg mL^−1^	-	<7.5	<15	[29]
Milk	Sex hormones	VALLME-MSPE	Choline chloride–Urea (1:2)	80–116	HPLC-DAD	0.1–50 µg mL^−1^	0.990	1–1.3 ng mL^−1^	2.5–4.5 ng mL^−1^	2–11	-	[51]
Milk	Synthetic hormones	Magnetic-assisted dispersive pipette-tip MSPE	Magnetic nanocube	34–74	HPLC-MS/MS	0.03–500 ng mL^−1^	0.996	0.01–0.02 ng mL^−1^	0.03–0.05 ng mL^−1^	-	-	[52]
CSF	E and DA	LLME	MIL	-	UHPLC-QqQ-MS/MS	-	-	-	-	-	-	[45]
Spinal cord	E, NE and DA	DLLME	MIL	-	UHPLC-QqQ-MS^2^	0.51–2360 µg g^−1^	0.995	0.15–0.64 µg g^−1^	0.5–2.1 µg g^−1^	2.1–6.8	3.7–8.0	[53]

DA, dopamine; DAD, diode array detection; DLLME, dispersive liquid–liquid microextraction; E, epinephrine (adrenaline); E2, estradiol; FLD, fluorescence detection; FSH, follicle-stimulating hormone; HPLC, high-performance liquid chromatography; LH, luteinizing hormone; LOD, limit of detection; LOQ, limit of quantification; LPME, liquid-phase microextraction; MEPS, microextraction by packed sorbent; MIL, magnetic ionic liquid; MS/MS, tandem mass spectrometry; MSPE, magnetic solid-phase extraction; NE, norepinephrine; QqQ, triple quadrupole; RSD, relative standard deviation; SPME, solid-phase microextraction; T, testosterone; TF, thin-film; UHPLC, ultra-high-performance liquid chromatography; VALLME, vortex-assisted liquid–liquid microextraction.

## Data Availability

No new data were created or analyzed in this study. Data sharing is not applicable to this article.

## Abbreviations

17-OHP, 17-hydroxyprogesterone; ABA, abscisic acid; CE, capillary electrophoresis; CNS, central nervous system; COF, covalent organic framework; CSF, cerebrospinal fluid; DA, dopamine; DAD, diode array detection; DES, deep eutectic solvent; DHEA, dehydroepiandrosterone; DHT, dihydrotestosterone; DLLME, dispersive liquid–liquid microextraction; DLLME-SFO, dispersive liquid–liquid microextraction with solidification of floating organic drop; E, epinephrine (adrenaline); E1, estrone; E2, estradiol; E3, estriol; EDC, endocrine-disrupting chemical; EE2, ethinylestradiol; EME, electromembrane extraction; FLD, fluorescence detection; FSH, follicle-stimulating hormone; GAC, green analytical chemistry; GC, gas chromatography; GH, growth hormone; GnRH, gonadotropin-releasing hormone; HFIP, hexafluoroisopropanol; HILIC, hydrophilic interaction chromatography; HPLC, high-performance liquid chromatography; HRMS, high resolution mass spectrometry; LC, liquid chromatography; LH, luteinizing hormone; LLE, liquid–liquid extraction; LOD, limit of detection; LOQ, limit of quantification; LPME, liquid-phase microextraction; MDL, method detection limit; MEPS, microextraction by packed sorbent; MIL, magnetic ionic liquid; MIL-LLME, magnetic ionic liquid-based liquid–liquid microextraction; MIP, molecularly imprinted polymer; MISPE, molecularly imprinted solid-phase extraction; MNPs, magnetic nanoparticles; MS, mass spectrometry; MS/MS, tandem mass spectrometry; MSPE, magnetic solid-phase extraction; MWCNTs, multi-walled carbon nanotubes; NADES, natural deep eutectic solvent; NE, norepinephrine; P4, progesterone; PDMS, polydimethylsiloxane; PGs, prostaglandins; PRL, prolactin; QqQ, triple quadrupole; RSD, relative standard deviation; rT3, reverse triiodothyronine; SBME, solvent bar microextraction; SBSE, stir bar sorptive extraction; SDME, single-drop microextraction; SPE, solid-phase extraction; SPME, solid-phase microextraction; SUPRAS, supramolecular solvent; T, testosterone; T2, diiodothyronine; T3, triiodothyronine; T4, thyroxine; TF, thin-film; THs; thyroid hormones; TOF, time of flight; UALLME, ultrasound-assisted liquid–liquid microextraction; UHPLC, ultra-high-performance liquid chromatography; VALLME, vortex-assisted liquid–liquid microextraction.
